# PremPS: Predicting the impact of missense mutations on protein stability

**DOI:** 10.1371/journal.pcbi.1008543

**Published:** 2020-12-30

**Authors:** Yuting Chen, Haoyu Lu, Ning Zhang, Zefeng Zhu, Shuqin Wang, Minghui Li

**Affiliations:** Center for Systems Biology, Department of Bioinformatics, School of Biology and Basic Medical Sciences, Soochow University, Suzhou, China; Koç University, TURKEY

## Abstract

Computational methods that predict protein stability changes induced by missense mutations have made a lot of progress over the past decades. Most of the available methods however have very limited accuracy in predicting stabilizing mutations because existing experimental sets are dominated by mutations reducing protein stability. Moreover, few approaches could consistently perform well across different test cases. To address these issues, we developed a new computational method PremPS to more accurately evaluate the effects of missense mutations on protein stability. The PremPS method is composed of only ten evolutionary- and structure-based features and parameterized on a balanced dataset with an equal number of stabilizing and destabilizing mutations. A comprehensive comparison of the predictive performance of PremPS with other available methods on nine benchmark datasets confirms that our approach consistently outperforms other methods and shows considerable improvement in estimating the impacts of stabilizing mutations. A protein could have multiple structures available, and if another structure of the same protein is used, the predicted change in stability for structure-based methods might be different. Thus, we further estimated the impact of using different structures on prediction accuracy, and demonstrate that our method performs well across different types of structures except for low-resolution structures and models built based on templates with low sequence identity. PremPS can be used for finding functionally important variants, revealing the molecular mechanisms of functional influences and protein design. PremPS is freely available at https://lilab.jysw.suda.edu.cn/research/PremPS/, which allows to do large-scale mutational scanning and takes about four minutes to perform calculations for a single mutation per protein with ~ 300 residues and requires ~ 0.4 seconds for each additional mutation.

## Author summary

The development of computational methods to accurately predict the impacts of amino acid substitutions on protein stability is of paramount importance for the field of protein design and understanding the roles of missense mutations in disease. However, most of the available methods have very limited predictive accuracy for mutations increasing stability and few could consistently perform well across different test cases. Here we present a new computational approach PremPS, which is capable of predicting the effects of single point mutations on protein stability. PremPS employs only ten evolutionary- and structure-based features and is trained on a symmetrical dataset consisting of the same number of cases of stabilizing and destabilizing mutations. Our method was tested against numerous blind datasets and shows a considerable improvement especially in evaluating the effects of stabilizing mutations, outperforming previously developed methods. PremPS is freely available as a user-friendly web server at http://lilab.jysw.suda.edu.cn/research/PremPS/, which is fast enough to handle the large number of cases.

This is a *PLOS Computational Biology* Methods paper.

## Introduction

Protein stability is one of the most important factors that characterize protein function, activity, and regulation [1]. Missense mutations can lead to protein dysfunction by affecting their stabilities and interactions with other biological molecules [2–9]. Several studies have shown that the mutations are deleterious due to decreasing or enhancing the stability of the corresponding protein [10–15]. To quantify the effects on protein stability requires estimating the changes in folding/unfolding Gibbs free energy induced by mutations. Experimental measurements of protein stability changes are laborious and appropriate only for proteins that can be purified [16]. Therefore, the computational prediction is urgently required, which would help the prioritization of potentially functionally important variants and become vital to many fields, such as medical applications [17] and protein design [18].

A lot of computational approaches have been developed in the last decades to predict the effects of single mutations on protein stability [19–48]. The vast majority of them are machine learning approaches and based on protein 3D structures. They are different in terms of algorithms used for building models, structural optimization procedures, or features of energy functions. The prediction performances of these methods have been assessed and compared using several different datasets of experimentally characterized mutants [14,49–52]. The results indicate that all methods showed a correct trend in the predictions but with inconsistent performances for different test sets. A majority of methods presented moderate or low accuracies when applied to the independent test sets, and INPS3D [47], PoPMuSiC [21], FoldX [28], and mCSM [22] that are among the most tested predictors showed relatively better performances in comparison with other methods on most of the data sets.

The machine learning approaches are prone to have overfitting problems [53], namely their predictions tend to be biased towards the characteristics of learning datasets. The training data sets available so far with experimentally determined protein stability changes are enriched with destabilizing mutations [21,54]. Thus, the vast majority of predictors that did not consider the unbalance of the training dataset showed a better performance for predicting destabilizing than stabilizing mutations [55,56]. A study constructed a balanced data set with an equal number of destabilizing and stabilizing mutations and was used to assess the performance of 15 methods [57]. The results showed that almost all these predictors present a strong bias towards predicting the destabilizing mutations. Additionally, two recent studies discussed the problem of bias of anti-symmetric property for six predictive methods [58,59]. The anti-symmetric property, namely, free energy change introduced by a forward mutation (ΔΔ*G*_*F*_) plus the change induced by its reverse mutation (ΔΔ*G*_*R*_) should be equal to zero. Correcting for such bias in the method’s performance is not a trivial task, which requires enriching the training set with stabilizing mutations and developing new energy functions. Several algorithms have been proposed to correct this bias, but the prediction accuracy has yet to be improved [46–48,57–59].

To address this issue, we developed PremPS that uses a novel scoring function composed of only ten features and trains on a balanced dataset including five thousand mutations, half of which belong to destabilizing mutations and the remaining half are stabilizing mutations. It has been comprehensively validated that PremPS performs significantly better than other methods especially in predicting the effects of stabilizing mutations and shows a very low prediction bias toward the anti-symmetric property. In addition, we further estimated the performance of our method on different types of structures using all available experimental structures of a protein and models built based on templates with different sequence identifies. The results demonstrate that our method performs well across different types of structures except for low-resolution structures and the models built based on templates with low sequence identity.

## Materials and methods

### Experimental dataset for parameterizing PremPS

ProTherm database is a collection of thermodynamic parameters for wild-type and mutant proteins [54]. It contains unfolding Gibbs free energy changes that provide important clues for estimating and interpreting the relationship among structure, stability, and function of proteins and their mutants. It is frequently used as a training template for developing *in silico* prediction approaches.

S2648 dataset includes 2,648 non-redundant unique single-point mutations from 131 globular proteins (S1A Fig), which was derived from the ProTherm database and compiled by [21]. Among these 131 proteins, there are 110 clusters of “similar proteins”. MMseqs2 software [60] was used to find the “similar proteins”; the sequence identity is set to 25% and the alignment covers at least 50% of query and target sequences. S2648 was used as the training dataset of PoPMuSiC [21], mCSM [22], DUET [23] and INPS3D [47] methods. Here, we also used the mutations and their unfolding Gibbs free energy changes from the S2648 to parameterize the PremPS model. The protein 3D structures were updated by applying the following criteria: structure obtained or extracted from monomer or homomer is preferred over heteromer; wild-type protein structure is preferred over mutant; structure with a minimal number of ligands is used; crystal structure is preferred over NMR; higher resolution structure is chosen, and the resolution of the crystal structure is 3 Å or higher. The multimeric state of each protein was either assigned by manually checking the references used to measure protein stability changes or retrieved from the PQS server [61].

Unfolding free energy change (ΔΔ*G*) of a system can be characterized as a state function where the ΔΔ*G*_*F*_ value of a forward mutation plus ΔΔ*G*_*R*_ of its reverse mutation should be equal to zero. Given the unbalanced nature of the S2648 dataset with 2,080 destabilizing (decreasing stability, ΔΔ*G*_*exp*_ ≥ 0) and 568 stabilizing (increasing stability, ΔΔ*G*_*exp*_ < 0) mutations, we modeled their reverse mutations in order to establish a more accurate computational method. Therefore, the final training set for parameterizing PremPS model contains 5,296 single mutations (it will be referred to as S5296) (S1A Table). The dataset is available for download from https://github.com/minghuilab/PremPS.

For the forward mutations, 3D structures of wild-type proteins were obtained from the Protein Data Bank (PDB) [62]. For the reverse mutations, the initial protein 3D structures were produced by the BuildModel module of FoldX [28] using wild-type protein structures as the templates. FoldX only optimizes the neighboring side chains around the mutation site when creating a mutant structure. We did not produce the mutant structures for either forward or reverse mutations.

### Experimental datasets used for testing

First, we used the following eight datasets that were taken from the previous studies to assess the predictive performance of PremPS and perform the comparison with other computational methods [21–48].

- S350, it is a randomly selected subset from S2648 including 350 mutations from 67 proteins compiled by [21]. This dataset is widely used to compare the performance of different methods. During the comparison, all methods were retrained after removing S350 from their training sets.
- S605, it was compiled from the Protherm database by [26] and contains 605 mutations from 58 proteins, which is the training dataset of Meta-predictor method [26].
- S1925, it includes 1,925 mutations from 55 proteins evenly distributed over four major SCOP structural classes, which is the training dataset of AUTOMUTE method [29].
- S134, it consists of experimentally determined stability changes for 134 mutations from sperm-whale myoglobin [49], and six different high-resolution crystal structures of myoglobin were used for the energy calculation.
- p53, it includes 42 mutations within the DNA binding domain of the protein p53 with experimentally determined thermodynamic effects, and the data was obtained from [22].
- S^sym^, a dataset was manually curated by [57]. It contains 684 mutations, half of which belong to forward mutations, and the remaining half are reverse mutations with crystal structures of the corresponding mutant proteins available.
- S250, it contains an equal number of forward and reverse mutations from nine proteins proposed by [58], for which both wild-type and mutant structures are available.
- S2000, it comprises 1,000 pairs of single-site mutations without experimental ΔΔ*G*_*exp*_ values [56]. The protein sequences for each pair differ by exactly one residue and the high-resolution protein 3D structures are available for all pairs. This dataset can be used to assess the bias of anti-symmetric property.

The number of mutations in each test set is shown in S1A and S1B Table, and the number of mutations in the training dataset of S5296 that overlaps with each test set and belongs to the “similar proteins” with more than 25% sequence identity to the proteins in each test set is presented in the S1C Table.

Next, we removed the redundant mutations from the above datasets and the overlapped mutations with S5296, then established a combined independent test set. S2000 was not used to construct this combined dataset due to the lack of ΔΔ*G*_*exp*_ values. The same criteria as the processing of the training dataset were used here to update the 3D structures of proteins. For the conflicting entries with multiple experimental measurements, if the difference between the maximal and minimal ΔΔ*G*_*exp*_ for this mutation is less than 1.0 kcal mol^-1^, we used the average value, otherwise we removed all entries (S1B Fig). As a result, the combined independent test set contains 921 single mutations from 54 proteins (it will be referred to as S921, S1A Table and S1C Fig). We further clustered these proteins according to the sequence identity of less than 25% and still obtained 47 protein clusters, which demonstrates the diversity of S921. S921 does not have the overlapped mutations with our training dataset, while it includes 41 “similar proteins” with more than 25% sequence identity to the proteins in the training set. All datasets are available for download from https://github.com/minghuilab/PremPS.

### The model of PremPS

The random forest (RF) regression scoring function of PremPS is composed of ten distinct features belonging to six categories (described below) and parameterized on the S5296 dataset. The contribution of each category of features is shown in the S2 Table.

- *PSSM* score is the Position-Specific Scoring Matrix created by PSI-BLAST. It finds similar protein sequences for the query sequence in which the mutation occurs by searching all protein sequences in NCBI non-redundant database, then builds a PSSM from the resulting alignment [63]. The default parameters were applied to construct PSSM profile.
- *ΔCS* represents the change of conservation after mutation calculated by PROVEAN method [64]. The features of *PSSM* and *ΔCS* illustrate that the evolutionarily conserved sites may play an important role in protein folding.
- *ΔOMH* is the difference of hydrophobicity scale between mutant and wild-type residue type. The hydrophobicity scale (OMH) for each type of amino acid residue, obtained from the study of [65], was derived by considering the observed frequency of amino acid replacements among thousands of related structures.
- *SASA*_*pro*_ and *SASA*_*sol*_ is the solvent accessible surface area (SASA) of the mutated residue in the protein and in the extended tripeptide respectively. The SASA of a residue in the protein and in the extended tripeptide was calculated by DSSP program [66] and obtained from [67], respectively.
- *P*_*FWY*_, *P*_*RKDE*_ and *P*_*L*_ is the fraction of aromatic residues (F, W or Y), charged residues (R, K, D or E), and leucine (L) buried in the protein core, respectively. For instance, ${P_{L}^{}}_{}^{}=\frac{N_{L}^{}}{N_{All}^{}}$, *N*_*L*_ is the number of all leucine residues buried in the protein core and *N*_*All*_ is the total number of residues. If the ratio of solvent accessible surface area of a residue in the protein and in the extended tripeptide is less than 0.2 [68], we defined this residue as buried in the core of the protein.
- *N*_*Hydro*_ and *N*_*Charg*_ is the number of hydrophobic (V, I, L, F, M, W, Y or C) and charged amino acids (R, K, D or E) at 23 sites centered on the mutated site in the protein sequence, respectively, [69].

In addition to Random Forest, we also tried two other popular learning algorithms of Support Vector Machine (SVM) and eXtreme Gradient Boosting (XGBoost), and the results shown in the S3 Table indicate that the random forest regression model presents the best performance.

The running time of PremPS for a single mutation per protein with ~ 300 residues is about four minutes, and it requires ~ 0.4 seconds for each additional mutation. Thus, it takes about ten minutes for PremPS to perform calculations for one thousand mutations introduced in the same protein. The source code of PremPS is publicly available at https://github.com/minghuilab/PremPS.

### Cross-validation procedures

We performed five types of cross-validation (CV1-CV5). For CV1 and CV2, we randomly chose 80% and 50% of mutations from the S5296 set respectively to train the model and used the remaining mutations for blind testing; the procedures were repeated 100 times. The number of mutations is not uniformly distributed over proteins (S1A Fig), in order to conquer the bias toward the proteins with the large number of mutations, we carried out the third type of cross-validation (CV3). Namely, a subset was created by randomly sampling up to 20 mutations for each protein from S5296; the procedure was repeated 10 times and resulted in 1,704 mutations in each subset. Then 80% of mutations were randomly selected from each subset to train the model and the rest of the mutations were used for testing, repeated 10 times. Next, we performed leave-one-protein-out validation (CV4), in which the model was trained on all mutations from 130 protein structures and the rest of the protein/mutations were used to evaluate the performance. This procedure was repeated for each protein and its mutations. Last, the leave-one-protein-cluster-out validation (CV5) was performed, where not only a protein in the validation set was removed from the training set, but also all other “similar proteins” with more than 25% sequence identity to this protein, repeated for each protein cluster. In all five described cross-validation procedures, during the training/test splits, the forward and their corresponding reverse mutations were retained in the same set, either training or testing.

### Statistical analysis and evaluation of performance

We used two measures of the Pearson correlation coefficient (R) and root-mean-square error (RMSE) to verify the agreement between experimental and predicted values of unfolding free energy changes. All correlation coefficients reported in the paper are significantly different from zero with p-value smaller than 0.01 (t-test). RMSE (kcal mol^-1^) is the standard deviation of the residuals (prediction errors). To check whether the difference in performance between PremPS and other methods is significant, we used Hittner2003 [70] and Fisher1925 [71] tests implemented in package *cocor* from R [72] to compare two correlation coefficients. Hittner2003 and Fisher1925 are used to compare two correlation correlations based on dependent groups with overlapping variable and independent groups, respectively. Receiver operating characteristics (ROC) curves were compared with the DeLong test [73].

To quantify the performance of different methods in distinguishing highly destabilizing (ΔΔ*G*_*exp*_ ≥ 1.0 kcal mol^-1^) or highly stabilizing (ΔΔ*G*_*exp*_ ≤ -1.0 kcal mol^-1^) mutations from the others, we performed Receiver Operating Characteristics (ROC) analyses. True positive rate is defined as TPR = TP/(TP+FN) and the false positive rate is defined as FPR = FP/(FP+TN) (TP: true positive; TN: true negative; FP: false positive; FN: false negative). In addition, the Matthews correlation coefficient (MCC) was calculated for estimating the quality of binary classification and accounting for imbalances in the labeled dataset:

$$MCC=\frac{TP*TN-FP*FN}{\sqrt{\left(TP+FP)(TP+FN)(TN+FP)(TN+FN\right)}}$$

To compare across methods, the maximal MCC value was reported for each method by calculating the MCC across a range of thresholds.

## Results and discussion

Currently, there are many published methods for predicting the protein stability change induced by a single mutation. However, they present diverse prediction performance for different test cases, and their accuracy has yet to be further improved to guide experimental research. Moreover, for the mutations increasing protein stability (stabilizing mutations), almost all of the methods show poor performance. Therefore, we developed the new approach of PremPS, in order to further improve the predictive performance for both destabilizing and stabilizing mutations and correct the predictive bias of anti-symmetric property.

PremPS employs only ten features belonging to six categories and is constructed by random forest regression algorithm implemented in the R randomForest package [74]. The number of trees “ntree” is set to 500 and the number of features, randomly sampled as candidates for splitting at each node, “mtry” value is set to 3. All features have a significant contribution to the model (S2 Table). The performance of PremPS trained and tested on S5296 is shown in S2A Fig and S4 Table. Pearson correlation coefficient between experimental and calculated unfolding free energy changes is 0.82 and the corresponding root-mean-square error and slope is 1.03 kcal mol^-1^ and 1.08 respectively.

### Performance on five types of cross-validation

**Fig 1:**
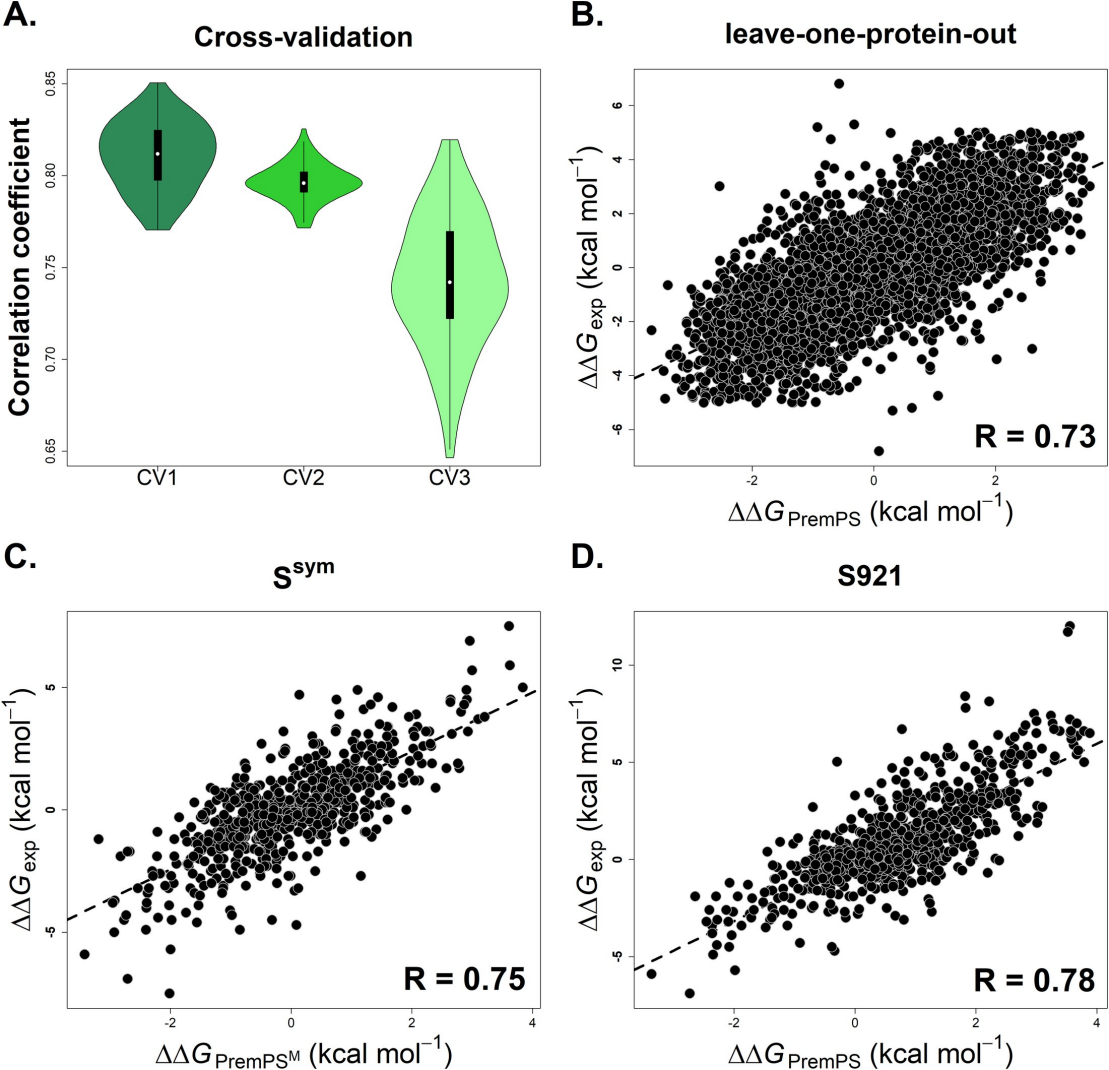
Pearson correlation coefficients between experimentally-determined and calculated values of changes in protein stability (ΔΔ*G*) for PremPS performing three types of cross-validation (A) and leave-one-protein-out validation on S5296 (B), and tested on the dataset of S^sym^ (C) and S921 (D), respectively. PremPS^M^: the model was retrained after removing the overlapped mutations including their corresponding reverse mutations with the dataset of S^sym^ from the training dataset. See also S2 Fig and S1 and S4 Tables.

Overfitting is one of the major concerns in machine learning, which may occur when the parameters are over-tuned to minimize the mean square deviations of predicted from experimental values in the training set. To overcome this problem, we performed five types of cross-validation (details were explained in the Methods section), which is capable of estimating the performance of a method on previously unseen data. As shown in Fig 1A and S4 Table, the correlation coefficient of each round in either CV1 or CV2 is higher than 0.77, and the mean values of R and RMSE for both validations are ~0.80 and ~1.08 kcal mol^-1^ respectively across the 100 rounds. Taking the bias that the distribution of the number of mutations over proteins is not uniform into account, the CV3 cross-validation was performed. The mean values of R and RMSE are 0.74 and 1.21 kcal mol^-1^ respectively for CV3. Moreover, we evaluated the performance of PremPS on two types of low redundant sets of proteins using leave-one-protein-out (CV4) and leave-one-protein-cluster-out validation (CV5), respectively, and the Pearson correlation coefficient reaches 0.73 and RMSE = 1.23 kcal mol^-1^ for both of them (Fig 1B and S4 Table).

One of the main features of our method is the usage of the symmetrical training dataset consisting of the same number of cases of forward and reverse mutations. Thus, we further classified the mutations into two categories: destabilizing versus stabilizing mutations and forward versus reverse mutations; the performance for each of them is shown in the S4 Table. PremPS shows the balanced prediction accuracy for the categories of destabilizing/stabilizing and forward/reverse mutations, although their correlation coefficients decrease compared to all mutations. For instance, R is 0.65, 0.62, 0.72, 0.69, and 0.81 for destabilizing, stabilizing, forward, reverse, and all mutations, respectively, upon CV1 cross-validation (S4 Table). Since the prediction method is quite successful in matching experimental data, the majority of forward mutations are in the first quadrant and reverse mutations are in the third quadrant, which results in an artificially high correlation coefficient for all mutations (S2A Fig). In addition, the correlation coefficient (R_RF_) between predicted ΔΔ*G* values of the forward and reverse mutations is ~ -0.90 indicating a very low-biased prediction of anti-symmetric property. In the S4 Table, we also present the performance for mutations occurring in protein core and surface, respectively. The definition of core and surface is according to the location of the mutated site in the protein 3D structure which has been illustrated in the Methods section. The experimental ΔΔ*G* values for the majority of surface mutations are distributed near zero (S4A Fig), which might be the reason for the relatively lower correlation coefficient and RMSE compared to the mutations in the core of the protein.

### Validation on the test sets

Eight widely used datasets were used to estimate the performance of PremPS and perform the comparison with other methods. Among them, three datasets of S^sym^, S250, and S2000 include pairs of forward and reverse mutations which can further be used to check the issue of bias of anti-symmetric property (ΔΔ*G*_*F*_+ΔΔ*G*_*R*_ = 0) (see Methods and S1B Table for more details). The performances of PremPS and different methods on these eight datasets are presented in S5A–S5H Table. Since the training dataset of S5296 includes the overlapped mutations and “similar proteins” with each test set (S1C Table), we retrained the model after removing the overlapped mutations including their corresponding reverse mutations (named as PremPS^M^) and all mutations in the “similar proteins” (named as PremPS^P^) from the training dataset, respectively, and then applied to each test set. The values of R and RMSE of all other methods were taken from the published papers directly, so the number of methods included in comparison with PremPS across the eight datasets is not consistent. To keep the comparison between PremPS and other methods equally and fairly, we also used the same protocol as the other methods to train PremPS and test on each dataset (the corresponding performance is shown in bold in the S5 Table). The results shown in Fig 1C and Tables 1 and S5 indicate that our method performs best or one of the best among all test cases, has the highest prediction accuracy for either forward or reverse mutations and a very low prediction bias of anti-symmetric property, and still shows robust performance even if PremPS^M^ or PremPS^P^ model was applied to each test set.

**Table 1:** The performance of PremPS^M^ tested on eight datasets. **Here, the PremPS model was retrained after removing the overlapped mutations including their corresponding reverse mutations with each test set from the training set**. The number of overlapped mutations is provided in the S1C Table.

Dataset	R	RMSE	R_FR_
S350	0.72	1.09	
S605	0.70	1.51	
S1925	0.59	1.48	
S134	0.65	0.84	
p53	0.72	1.47	
S^sym^	0.75	1.26	-0.91
S250	0.78	1.22	-0.92
S2000			-0.92

R: Pearson correlation coefficient between experimental and predicted ΔΔ*G* values. RMSE (kcal mol^-1^): root-mean square error. R_FR_: Pearson correlation coefficient between predicted ΔΔ*G* values of the forward and reverse mutations. All correlation coefficients shown in the table are statistically significantly different from zero (p-value < 0.01, t-test).

Moreover, we removed the redundant mutations from the above datasets and the overlapped mutations with the training set of S5296 and established the independent test set of S921 (details were explained in the Methods section). For all mutations in this dataset, we calculated the values of stability changes using PremPS and four other methods of INPS3D [47], PoPMuSiC [21], FoldX [28], and mCSM [22] that were among the most tested and reliable predictors (see S5 Table). The results reported in Figs 1D and S2B and Table 2 demonstrate that PremPS achieves the highest prediction accuracy especially for stabilizing mutations with R of up to 0.78, 0.72, and 0.60 for all, destabilizing and stabilizing mutations respectively. However, there are 16 mutations with a large difference of more than 4 kcal mol^-1^ between experimental and predicted values (see Figs 1D and S5A). It can be seen from S5B Fig that the experimental values of these mutations are all greater than 5 kcal mol^-1^ except one on the bottom line, while in our training dataset, the experimental values of all mutations are less than 5 kcal mol^-1^. This is probably why the experimental and predicted values for these 16 mutations differ so much. Furthermore, we evaluated the performance of PremPS when trained only on the forward mutation dataset of S2648 and tested on the S921. The correlation coefficient is 0.73 and 0.29 for destabilizing and stabilizing mutations, respectively (S6 Table). The results confirm that the usage of reverse mutations improved the performance of our model in estimating the effects of stabilizing mutations without compromising the prediction accuracy for destabilizing mutations. Next, we excluded the mutations in the “similar proteins” with more than 25% sequence identity to the proteins in S921 from the training set (The number of mutations is 3710, S1C Table), retrained the model and tested it on the S921. The PremPS still presents a good performance with R = 0.70 (PremPS^P^ in the S6 Table).

**Table 2:** Comparison of methods’ performance on the independent test set of S921.

Method	All mutations		Destabilizing		Stabilizing	
	R	RMSE	R	RMSE	R	RMSE
PremPS	0.78	1.48	0.72	1.54	0.60	1.33
INPS3D	0.68	1.62	0.64	1.61	0.38	1.64
PoPMuSiC	0.64	1.68	0.68^#^	1.48	-	2.06
FoldX	0.57	2.06	0.56	1.99	0.22	2.21
mCSM	0.52	1.85	0.57	1.63	-	2.25

R: Pearson correlation coefficient between experimental and predicted ΔΔ*G* values. RMSE (kcal mol^-1^): root-mean square error. The number of destabilizing (ΔΔ*G*_*exp*_ ≥ 0) and stabilizing (ΔΔ*G*_*exp*_ < 0) mutations in S921 is 634 and 287, respectively (S1 Table). Only correlation coefficients with statistically significantly different from zero (p-value < 0.01, t-test) are shown. The differences in R between PremPS and other methods are significant (all p-values << 0.01 compared to PremPS except #p-value = 0.04, Hittner2003 test).

**Fig 2:**
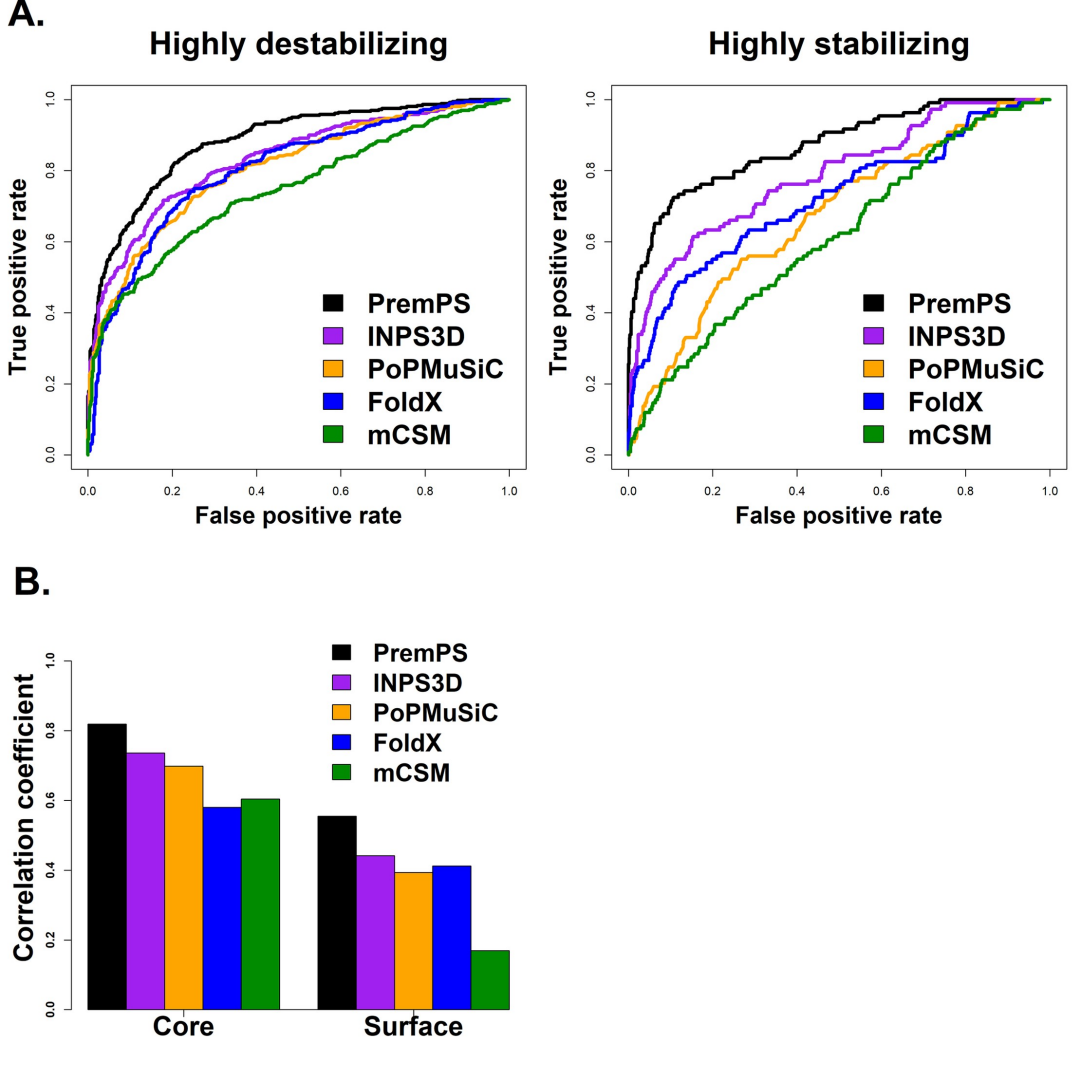
Comparative performance of PremPS and four other methods of INPS3D, PoPMuSiC, FoldX, and mCSM on the independent test set of S921. (A) ROC curves for predicting highly destabilizing (ΔΔ*G*_*exp*_ ≥ 1 kcal mol^-1^) and highly stabilizing mutations (ΔΔ*G*_*exp*_ ≤ -1 kcal mol^-1^). PremPS has substantially higher AUC-ROC than other methods (p-value < 0.01, DeLong test, S3B Fig). (B) Pearson correlation coefficients between predicted and experimental ΔΔ*G* for mutations occurring in protein core and surface. The difference in R between PremPS and other methods is significant (p-value < 0.01, Hittner2003 test). More details are shown in S3 and S4 Figs.

In addition, we carried out the ROC analysis in order to quantify the performance of PremPS in distinguishing highly destabilizing (ΔΔ*G*_*exp*_ ≥ 1.0 kcal mol^-1^) and highly stabilizing (ΔΔ*G*_*exp*_ ≤ -1.0 kcal mol^-1^) mutations from the others. Figs 2A and S3 show the excellent performance of PremPS in evaluating highly destabilizing/stabilizing mutations, outperforming four other methods. Besides, PremPS performed well for both core and surface mutations (Figs 2B and S4 and S6 Tables).

### How transferable is PremPS across different structures?

In the above study, we selected a single structure for a protein following the criteria shown in the Methods section to calculate the stability change. As we know, a protein could have multiple structures available, and if another structure of the same protein is used, the predicted change in stability for structure-based methods might be different. Therefore, we will further estimate the impact of using different structures on prediction accuracy. The forward mutation dataset of S2648 and the independent test set of S921 were used to carry out this analysis. Since the prediction accuracy for mutations at the protein-protein interface of homomers from S2648 is reduced when using monomers (results are shown in S7A and S8 Tables), in order to avoid interference from such mutations, the analysis was further restricted to mutations in monomeric protein structures. In the dataset of S921, all structures are in a monomeric state. Next, 2297 mutations in S2648 and 824 mutations in S921 can be mapped to multiple protein structures (S7B Table). Therefore, the four datasets, named S2297, S824, RS2297, and RS824, were used to assess the predictive performance of PremPS across different structures. S2297 and S824, subsets of S2648 and S921 respectively, consist of a selected single one structure for a protein, while the datasets of RS2297 and RS824 include all the other mapped redundant structures. We then applied PremPS trained on S5296 to these four datasets. For S2297 and RS2297, the leave-one-protein-out validation (CV4) results were also provided. The 131 models, generated when performing the CV4 validation on the dataset of S5296 (see Methods for more details), were used to produce the CV4 results for RS2297.

First, we analyzed the effects of different protein structures on the derived prediction for changes in stability. PremPS is a structure-based method and could produce different predicted values when using different structures, but the differences of ΔΔ*G*_*PremPS*_ calculated by different structures for the majority of mutations are relatively small with a standard deviation of less than 0.5 kcal mol^-1^ (S6A Fig). The predictive performance of using all the other redundant structures is not significantly different from that of using selected single one structures (Tables 3 and S9), and the correlation coefficient between predicted stability changes for S2297/S824 and the mean values for RS2297/RS824 is very high (R ~ 1.0, S6B Fig). Although we cannot afford which structure will give the best prediction for a special case, from the above statistical analysis, it can be concluded that our method is robust and its predictive accuracy is not affected strongly by using different structures.

**Table 3:** PremPS’ performance on four datasets. Leave-one-protein-out validation (CV4) results are shown for S2297 and RS2297. More details are provided in S9 Table.

Dataset	R	RMSE
S2297	0.57	1.23
RS2297	0.59	1.23
S824	0.74	1.48
RS824	0.71	1.61

R: Pearson correlation coefficient between experimental and predicted ΔΔ*G* values. RMSE (kcal mol^-1^): root-mean square error. No statistically significantly differences in correlation coefficient are observed between S2297 and RS2297, and S824 and RS824 (Fisher1925 test).

Second, we analyzed the differences in prediction performance of PremPS across protein structures resolved by distinct experimental methods and at different resolutions. The number of mutations and protein structures resolved by the experimental method of X-ray, NMR or Cryo-EM and two or more methods in the datasets of S2297, S824, RS2297 and RS824, respectively, are shown in S7C Table, and the corresponding performance of PremPS for each category is shown in S10 Table. In general, PremPS performs well in structures resolved by X-ray, NMR or Cryo-EM method. By comparing the performance for the cases resolved by two or more methods, we found that PremPS performs better in X-ray than NMR structures and in Cryo-EM than X-ray structures for the cases from RS2297 and RS824, respectively. Next, for the structures resolved by X-ray and Cryo-EM, we analyzed how performance scale is as a function of the resolution of protein structures. According to the distribution of resolution (S6C Fig), we classified the structures into two categories: ≤ 3Å and > 3Å, and ≤ 2Å, 2Å ~ 3Å, 3Å ~ 4Å and > 4Å (The number of mutations and protein structures at different resolutions is shown in S7D Table). The results reported in the S11 Table indicate that the prediction accuracy decreases with the decrease of structural resolution. The resolution of 3Å can be used as a threshold for protein design studies.

The monomeric protein structures used in our study were either resolved in a monomeric state or extracted from homomers and heteromers (S7E Table). As can be seen from the S12 Table, proteins that were determined in a monomeric state hold a slightly higher prediction accuracy compared to those derived from homomers or heteromers.

Currently, more and more structures of homomeric and heteromeric complexes of high molecular weight were determined by Cryo-EM experiments. These structures have not yet been used or put to test for structure-based design in a quantitative manner. In our study, the datasets of RS2297 and RS824 contain 14 mutations from three proteins that can be mapped to high molecular weight Cryo-EM structures (more than 800kDa). S7 Fig shows their predicted values using X-ray/NMR structures and the structures extracted from high molecular weight Cryo-EM structures, respectively, and X-ray/NMR structures hold a slightly better performance than Cryo-EM for four mutations. However, a statistically significant conclusion cannot be drawn because of the small amount of data.

Last, we investigated how errors in protein structure modeling affect prediction performance. Modeller software (version 9.25) [75] was used to identify potential templates in the ranges of sequence identity of 20–30%, 30–40%…, 90–100%, and build 3D models for each protein from S2297 and S824 datasets. The alignment should cover at least 85% of the target sequence length. The best model for each target-template pair was selected based on the molpdf score implemented in the Modeller [76]. For each range of sequence identity, the number of proteins for which at least one template were found and the number of modeled structures is given in the S7F Table. Then we calculated the stability changes using all modeled structures. Compared with experimental structures used, the prediction accuracy of PremPS is reduced significantly when using models built based on templates with a low sequence identity of less than 30% (Tables 4 and S13). In addition, the root-mean-square deviation (RMSD) between coordinates of all Cα atoms of experimental and modeled structures was used to measure the quality of the models. As can be seen from S8 Fig, most of the models have low deviations from the experimental structures. We further classified the models according to the ranges of RMSD: ≤ 3Å, 3Å-5Å, 5Å-10Å, and >10Å (S7G Table). The performance presented in the S14 Table indicates that the lower the quality of the model, the less accurate the prediction.

**Table 4:** Pearson correlation coefficient between experimental and predicted ΔΔ*G* values calculated using experimental and modeled structures in different ranges of sequence identity. Selected models: when several templates were available in a given range of sequence identity, the one whose sequence identity with the target was closest to the middle of the range and deviation from the experimental structure was the lowest was selected. Leave-one-protein-out validation (CV4) results are shown for S2297 and RS2297. More details are provided in S13 Table.

Dataset	Structure	20–30%	30–40%	40–50%	50–60%	60–70%	70–80%	80–90%	90–100%
S2297	Exp. Structs.	0.62	0.58	0.57	0.55	0.56	0.58	0.53	0.57
	All models	0.49*	0.53	0.54	0.50	0.56	0.60	0.57	0.53
	Selected models	0.56	0.55	0.56	0.55	0.54	0.57	0.53	0.58
S824	Exp. Structs.	0.72	0.68	0.69	0.57	0.72	0.74	0.70	0.74
	All models	0.65	0.67	0.71	0.57	0.67	0.69	0.68	0.76
	Selected models	0.55*	0.58	0.66	0.57	0.74	0.72	0.66	0.72

*p-value < 0.01 compared to experimental structures (Fisher1925 test).

### Online webserver

#### Input

**Fig 3:**
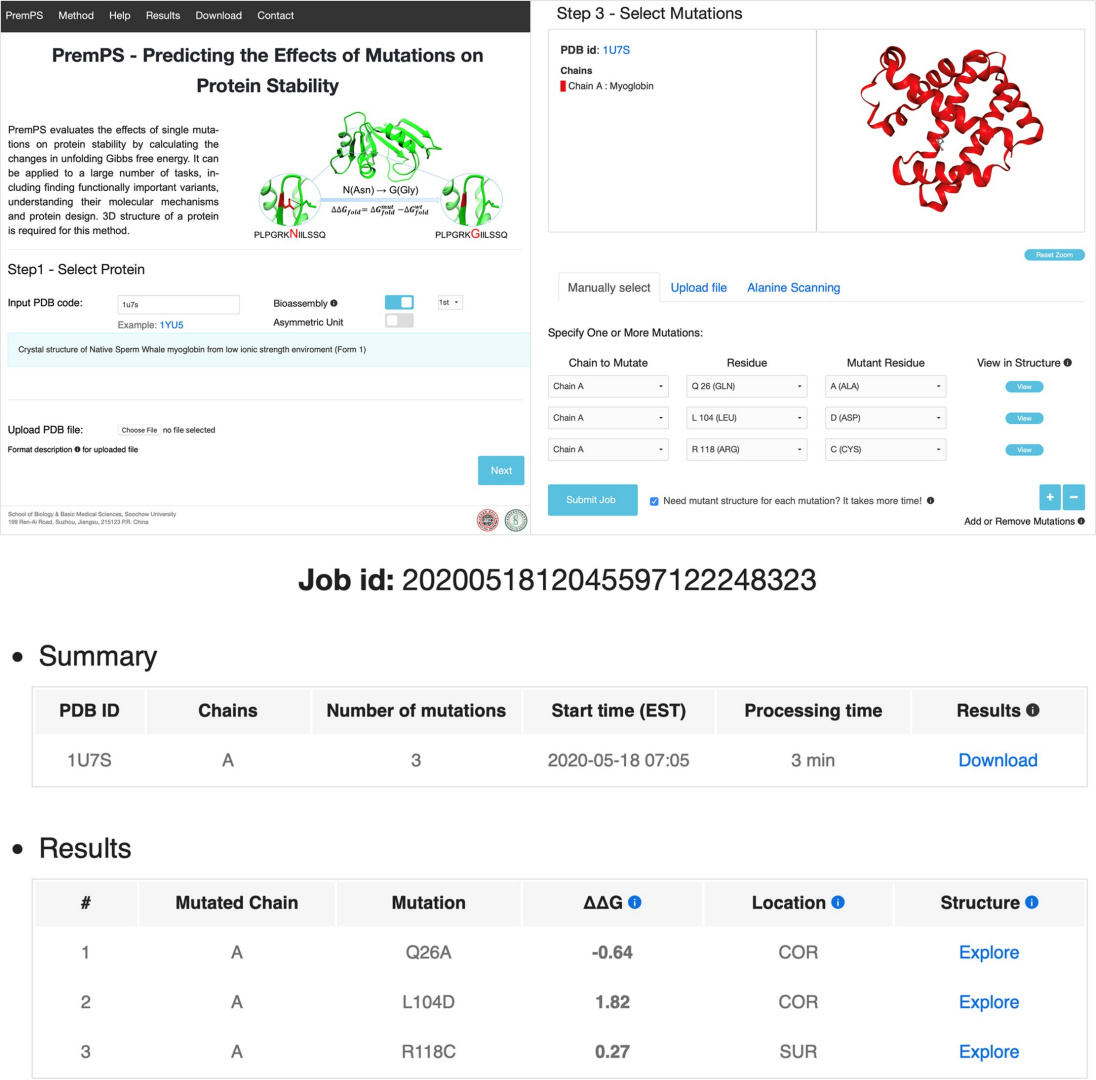
Left corner: the entry page of PremPS server; right corner: the third step for selecting mutations and three options are provided: “Specify One or More Mutations Manually”, “Upload Mutation List” and “Alanine Scanning for Each Chain”, see also S9 Fig; and bottom: final results, see also S10 Fig. “Processing time” refers to the running time of a job without counting the waiting time in the queue. The contribution of each feature is provided in the download file.

The 3D structure of a protein is required by the webserver, and the user can provide the Protein Data Bank (PDB) code or upload the coordinate file. When the user provides the PDB code, biological assemblies or asymmetric unit can be retrieved from the Protein Data Bank (Figs 3 and S9A). After the structure is retrieved correctly, the server will display a 3D view colored by protein chains and list the corresponding protein name (S9B Fig). At the second step, one or multiple chains that must belong to one protein can be assigned to the following energy calculation. The third step is to select mutations and three options are provided: “Upload Mutation List”, “Alanine Scanning for Each Chain” and “Specify One or More Mutations Manually” (Figs 3 and S9C). “Upload Mutation List” allows users to upload a list of mutations for large-scale mutational scans. “Alanine Scanning for Each Chain” allows users to perform alanine scanning for each chain. In the option of “Specify One or More Mutations Manually”, users can not only perform calculations for specified mutations but also be allowed to view the mutated residues in the protein structure.

#### Output

For each mutation of a protein, the PremPS server provides the following results (Figs 3 and S10): ΔΔ*G* (kcal mol^-1^), predicted unfolding free energy change induced by a single mutation (positive and negative sign corresponds to destabilizing and stabilizing mutations, respectively); location of the mutation (COR: core or SUR: surface), a residue is defined as buried in the protein core if the ratio of solvent accessible surface area of this residue in the protein and in the extended tripeptide is less than 0.2, otherwise it is located on the surface of the protein. In addition, for each mutation, our server outputs the contribution of each feature in the target function and provides an interactive 3D viewer showing the non-covalent interactions between the mutated site and its adjacent residues, generated by Arpeggio [77] (S10B Fig). The mutant structure is produced for each mutation upon the user’s request in the third step (S10C Fig). Usually, PremPS requires additional ~ 40 seconds to produce a mutant structure for a protein with ~ 300 residues.

## Supporting information

S1 Fig (A) The number of mutations for each protein structure in S2648 dataset. (B) The distribution of the differences between maximal and minimal experimentally-determined stability changes (ΔΔ*G*_max_—ΔΔ*G*_min_) for 232 mutations from datasets of S1925, S605, S^sym^ and S134 with multiple experimental measurements. Among them, the values of ΔΔ*G*_max_—ΔΔ*G*_min_ of 205 mutations are less than 1.0 kcal mol^-1^, which were included in the S921 dataset and the average value was used for each mutation. (C) The independent test set of S921 is composed of five datasets.
(PDF)

S2 Fig Pearson correlation coefficients between experimental and calculated values of changes in protein stability (ΔΔ*G*) for PremPS trained and tested on S5296 and performing the leave-one-protein-out validation. Black: forward mutations; Red: reverse mutations (A), and for INPS3D, PoPMuSiC, FoldX and mCSM methods tested on S921, respectively (B).
(PDF)

S3 Fig ROC analysis for predicting highly destabilizing and stabilizing mutations. (A) ROC curves for PremPS trained and tested on S5296 and applying leave-one-protein-out validation (CV4) on S5296. (B) AUC and MCC values for different methods tested on S921. The difference of AUC between PremPS and other methods is significant (p-value << 0.01, DeLong test). Maximum Matthews correlation coefficient is calculated for each method. (C) The definition and the number of mutations for making ROC curves.
(PDF)

S4 Fig (A) Distribution of experimental values of stability changes for mutations occurring in protein core and surface respectively. (B) Pearson correlation coefficients between experimental and calculated ΔΔ*G* values. The difference in R between PremPS and other methods is significant (p-value < 0.01, Hittner2003 test). (C) The number of core and surface mutations in S5296 and S921 datasets.
(PDF)

S5 Fig (A) Distribution of differences between experimental and predicted values for S921. There are 16 mutations with a large difference (ΔΔ*G*_exp_-ΔΔ*G*_PremPS_) of more than 4 kcal mol^-1^. (B) Experimental (exp) and predicted values (PremPS) in change of stability for these 16 mutations.
(PDF)

S6 Fig (A) Distribution of mean value (MV) and standard deviation (SD) of ΔΔ*G*_*PremPS*_ for mutations in datasets of RS2297and RS824. The mean value and standard deviation were calculated using all mapped structures of a protein. (B) Pearson correlation coefficients between ΔΔ*G*_*PremPS*_ calculated using selected single one structure for a protein in S2297/S824 and the mean value calculated using all other mapped redundant structures in RS2297/RS824. (C) Distribution of resolution of protein structures resolved by X-ray and Cryo-EM. Leave-one-protein-out validation (CV4) results were shown for S2297 and RS2297 datasets.
(PDF)

S7 Fig Experimental and predicted stability changes for 14 mutations from three proteins. One X-ray and two NMR structures from S2297 and S824 and three structures extracted from high molecular weight Cryo-EM structures (more than 800kDa) from RS2297 and RS824 were used to perform the calculations. Leave-one-protein-out validation (CV4) results are shown for S2297 and RS2297 datasets. The predicted stability changes of four mutations have a relatively large difference of ~ 0.5 kcal mol^-1^ between using X-ray/NMR structures and two high molecular weight Cryo-EM structures.
(PDF)

S8 Fig (A) Distribution of root-mean-square deviation (RMSD) between coordinates of all Cα atoms of experimental and modeled structures. (B) Boxplots of RMSD for different ranges of sequence identity of 20–30%, 30–40%…, 90–100%. The red line is 3Å.
(PDF)

S9 Fig (A) The entry page of PremPS server. (B) The second step for selecting protein chains. (C) The third step for selecting mutations and three options are provided: “Specify One or More Mutations Manually”, “Upload Mutation List” and “Alanine Scanning for Each Chain”. The mutant structure is produced for each mutation upon the user’s request in the third step.
(PDF)

S10 Fig (A) The final results. “Processing time” refers to the running time of a job without counting the waiting time in the queue. The contribution of each feature is provided in the download file. (B) Interactive 3D viewer showing the non-covalent interactions between the mutated site in the protein myoglobin (PDB ID: 1U7S, mutation: L104D) and its adjacent residues in the wild type (left) and mutant (right) respectively, generated by Arpeggio. The mutant structure was produced for each mutation for this job.
(PDF)

S1 Table Experimental datasets used for training and testing. (A) The number of mutations and proteins/structures in each dataset. (B) The number of forward and reverse mutations in the dataset of S^sym^, S250 and S2000, respectively. (C) The number of mutations and protein structures (in bracket) in the training dataset of S5296 that overlaps with each test set (the first row) and belongs to the “similar proteins” with more than 25% sequence identity to the proteins in each test set (the second row).
(PDF)

S2 Table The importance of each category of features for PremPS model. IncNodePurity is used for describing the importance which is the total decrease in node impurities from splitting on the variable, averaged over all trees.
(PDF)

S3 Table The performance on S5296 and S921 when the model was built using Random Forest (RF), Support Vector Machine (SVM) and eXtreme Gradient Boosting (XGBoost) algorithms respectively. Leave-one-protein-out validation (CV4) results were shown for S5296.
(PDF)

S4 Table The performance for PremPS trained and performing five types of cross-validation (CV1-CV5) on S5296 set. (PDF)

S5 Table Comparative performance of different methods on the dataset of S350. (A), S605 (B), S1925 (C), S134 (D), p53 (E), S^sym^ (F), S250 (G) and S2000 (H), respectively. The values of R and RMSE for all methods except PremPS were taken from the published papers directly. The performance of PremPS using the same protocol as the other methods when applied to each dataset is shown in bold. In addition, we provided the performance of PremPS^M^ and PremPS^P^. PremPS^M^: the model was retrained after removing the overlapped mutations and their corresponding reverse mutations with each test set from the training dataset; PremPS^P^: the model was retrained after removing all mutations in the “similar proteins” from the training dataset. The number of mutations removed were provided in the S1C Table.
(PDF)

S6 Table Comparison of methods’ performance on the independent test set of S921. PremPS: the model was trained on S5296; PremPS^F^: the model was trained on the forward mutation dataset of S2648; PremPS^P^: the model was retrained after removing all mutations in the “similar proteins” with more than 25% sequence identity to the proteins in S921 from the training dataset.
(PDF)

S7 Table The number of protein structures and mutations. (A) The number of mutations from monomeric and homomeric protein structures in S2648 dataset, respectively. The number of protein structures are shown in parentheses. Interface: mutations at the protein-protein interface of homomers. (B) The number of monomeric protein structures/mutations that can be mapped to multiple experimental structures in S2648 and S921, respectively. The number of mapped redundant structures has excluded the selected structures used in the datasets of S2648 and S921. (C) The number of protein structures resolved by experimental method of X-ray, NMR or Cryo-EM and two or three methods. (D) The number of protein structures resolved at different resolutions. (E) The number of protein structures resolved in a monomeric state or extracted from homomers and heteromers. (F) The number of proteins for which at least one templates were found and the number of modeled structures in each range of sequence identity. (G) The number of proteins and modeled structures in each range of root-mean-square deviation.
(PDF)

S8 Table The performance for PremPS applied on mutations from homomeric protein structures in S2648 and monomeric structures extracted from the corresponding homomers respectively. (PDF)

S9 Table Method’ performance on four datasets. S2297 and S824, subsets of S2648 and S921 respectively, consist of selected single one structure for a protein, while the datasets of RS2297 and RS824 include all the other mapped redundant structures.
(PDF)

S10 Table Prediction performance for protein structures resolved by experimental method of X-ray, NMR or Cryo-EM and two or three methods respectively. The number of proteins resolved by both NMR and Cryo-EM are almost the same as that resolved by X-ray, NMR and Cryo-EM (see S7C Table), so the performance for two methods of NMR and Cryo-EM is not shown.
(PDF)

S11 Table Prediction performance for protein structures resolved at different resolutions. (PDF)

S12 Table Prediction performance for protein structures resolved in the monomeric state or extracted from homomers and heteromers. (PDF)

S13 Table Prediction performance for models in different ranges of sequence identity. (PDF)

S14 Table Performance for models at different ranges of root-mean-square deviation (RMSD). (PDF)
