# Supplementary figures and images for: PremPS: Predicting the impact of missense mutations on protein stability
Source: PLoS Comput Biol. 2020 Dec 30;16(12):e1008543. doi: 10.1371/journal.pcbi.1008543 (PMC7802934; doi:10.1371/journal.pcbi.1008543)

A.

## The number of single mutations for each protein structure

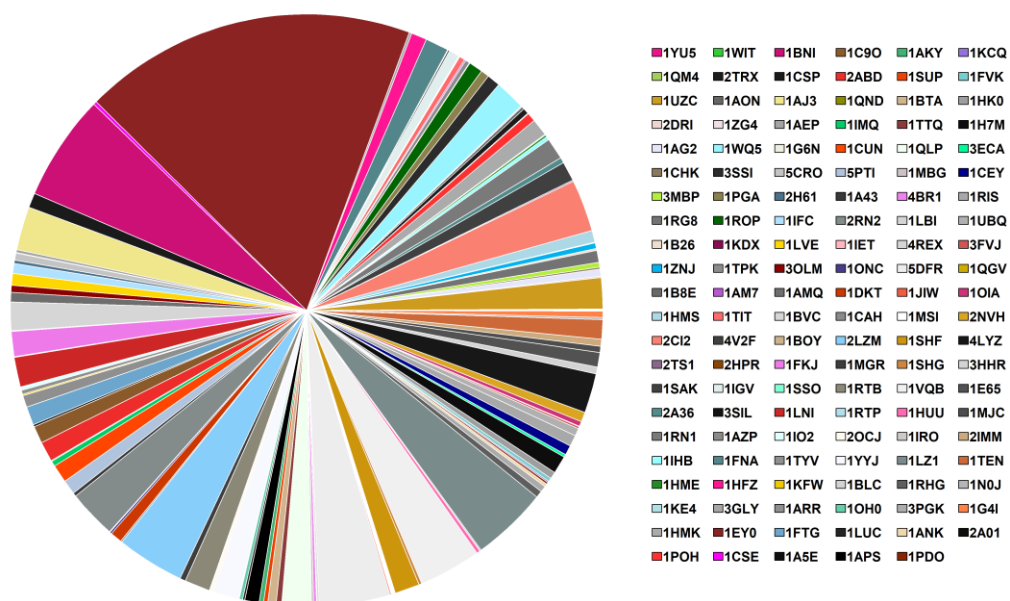

B.

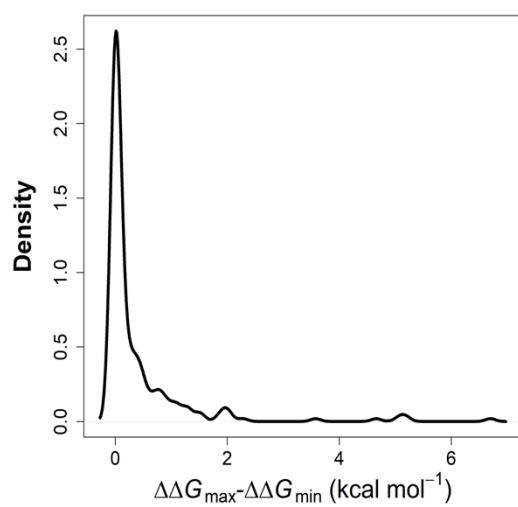

C.

## The composition of S921

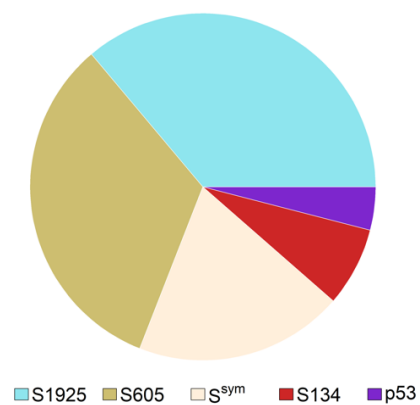

Supplement: S1 Fig — (A) The number of mutations for each protein structure in S2648 dataset. (B) The distribution of the differences between maximal and minimal experimentally-determined stability changes (ΔΔGmax—ΔΔGmin) for 232 mutations from datasets of S1925, S605, Ssym and S134 with multiple experimental measurements. Among them, the values of ΔΔGmax—ΔΔGmin of 205 mutations are less than 1.0 kcal mol-1, which were included in the S921 dataset and the average value was used for each mutation. (C) The independent test set of S921 is composed of five datasets. (PDF) [file pcbi.1008543.s001.pdf]

### A. Test on S5296

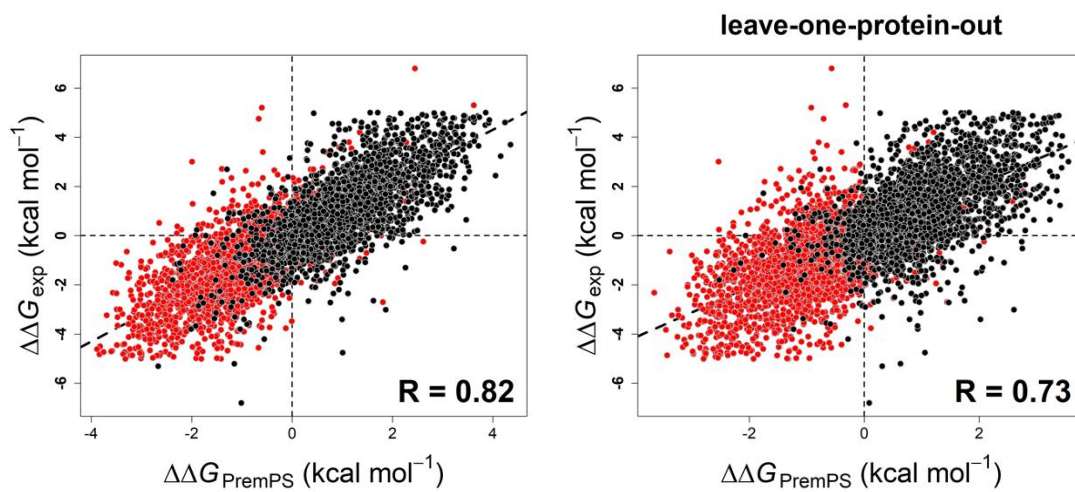

### B. Test on S921

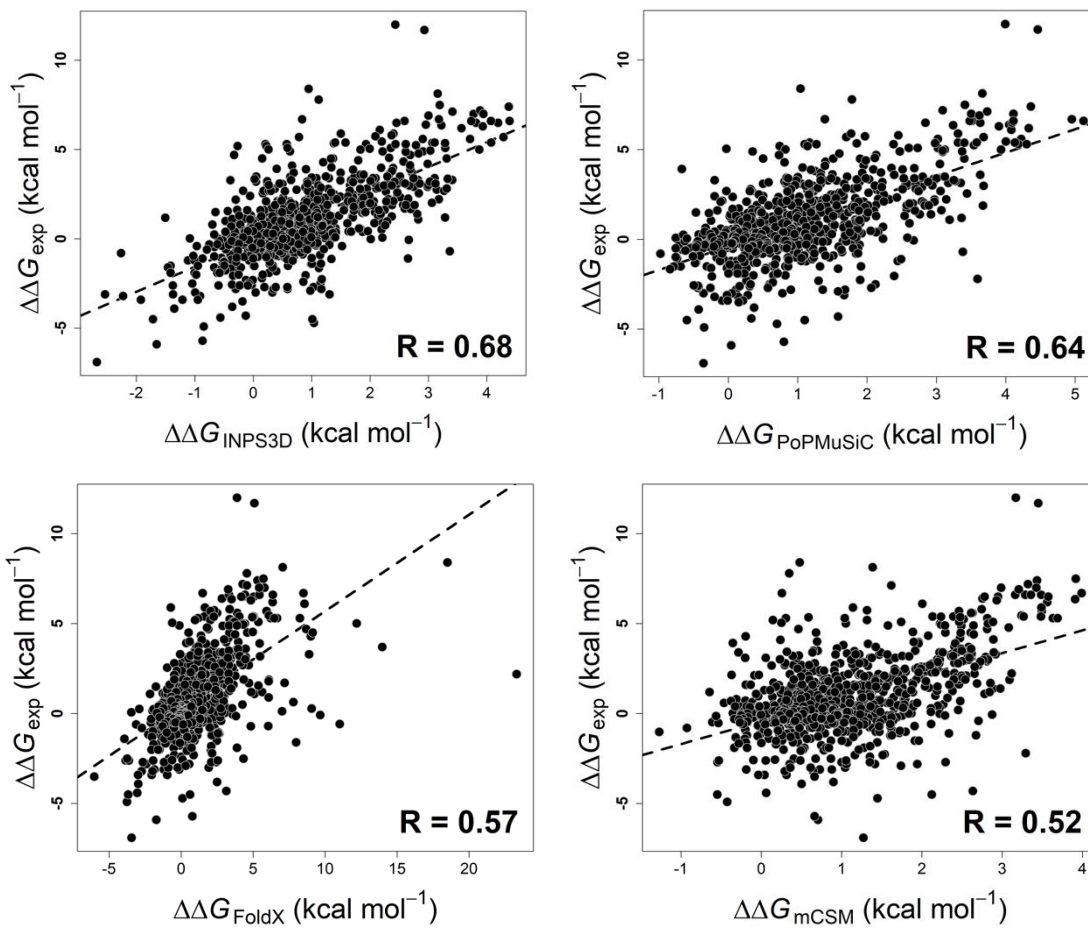

Supplement: S2 Fig — Black: forward mutations; Red: reverse mutations (A), and for INPS3D, PoPMuSiC, FoldX and mCSM methods tested on S921, respectively (B). (PDF) [file pcbi.1008543.s002.pdf]

**A.**

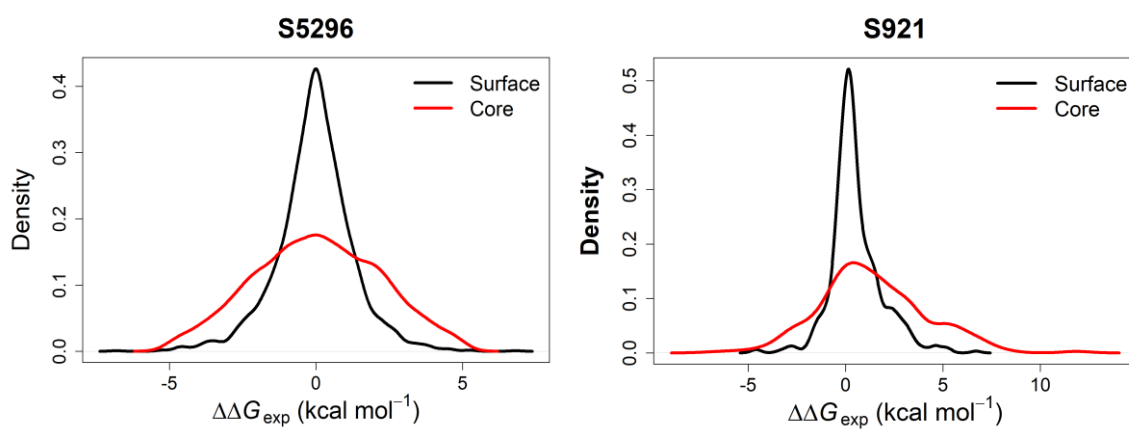

**B.**

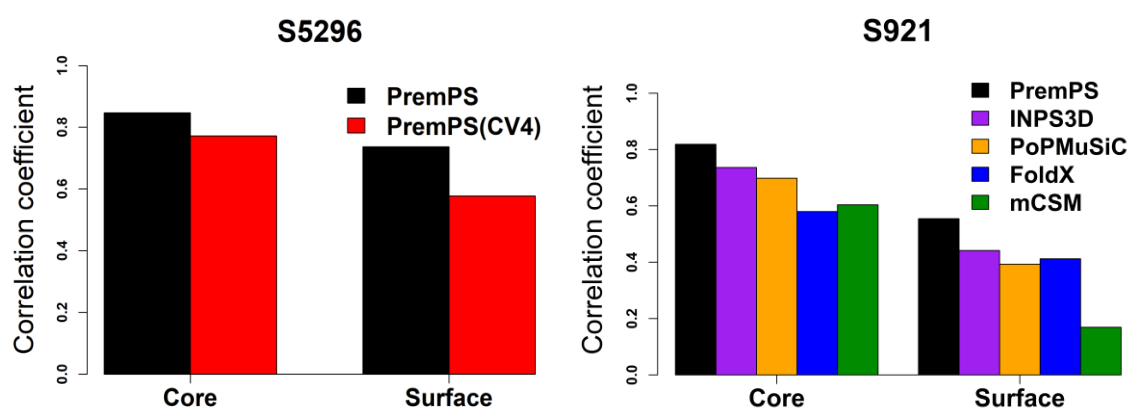

**C. The number of mutations**

| Category | S5296 | S921 |
|----------|-------|------|
| Core     | 2706  | 430  |
| Surface  | 2590  | 491  |

Supplement: S4 Fig — (A) Distribution of experimental values of stability changes for mutations occurring in protein core and surface respectively. (B) Pearson correlation coefficients between experimental and calculated ΔΔG values. The difference in R between PremPS and other methods is significant (p-value < 0.01, Hittner2003 test). (C) The number of core and surface mutations in S5296 and S921 datasets. (PDF) [file pcbi.1008543.s004.pdf]

**A.**

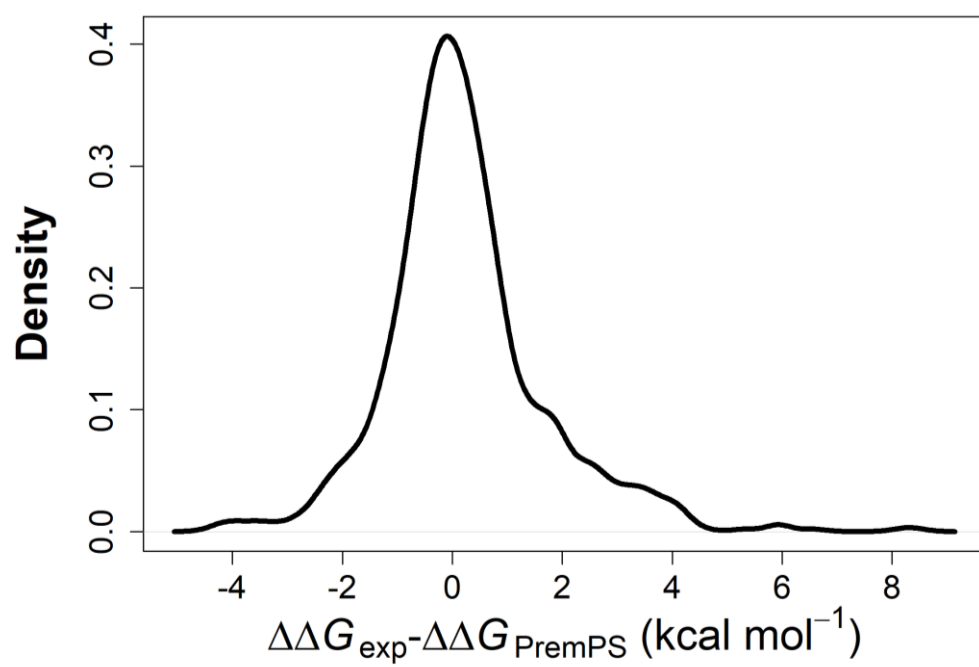

**B.**

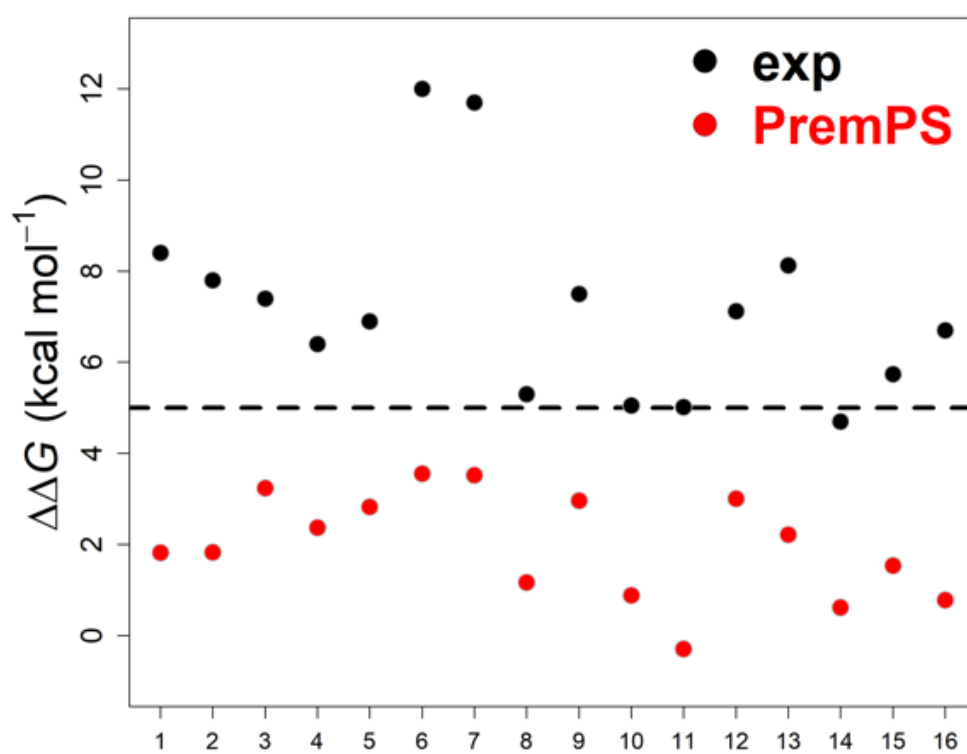

Supplement: S5 Fig — (A) Distribution of differences between experimental and predicted values for S921. There are 16 mutations with a large difference (ΔΔGexp-ΔΔGPremPS) of more than 4 kcal mol-1. (B) Experimental (exp) and predicted values (PremPS) in change of stability for these 16 mutations. (PDF) [file pcbi.1008543.s005.pdf]

A.

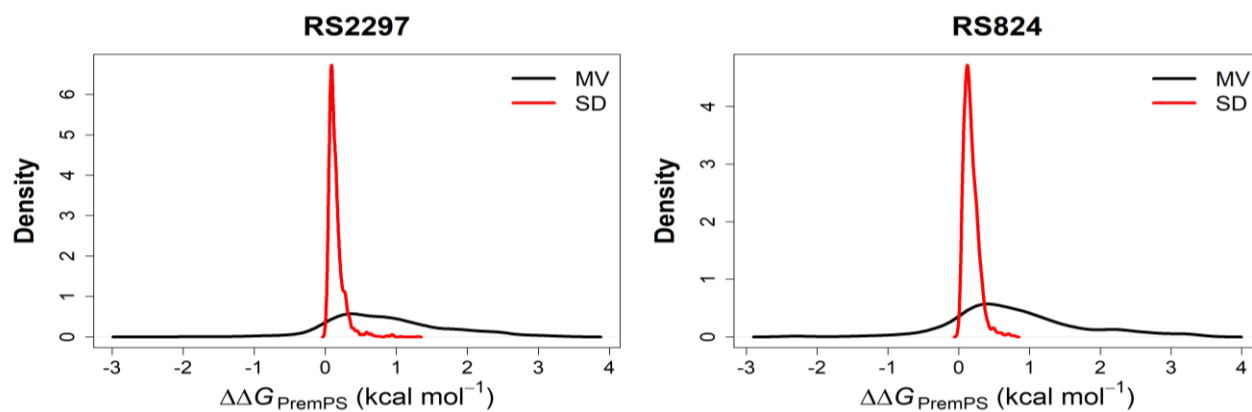

B.

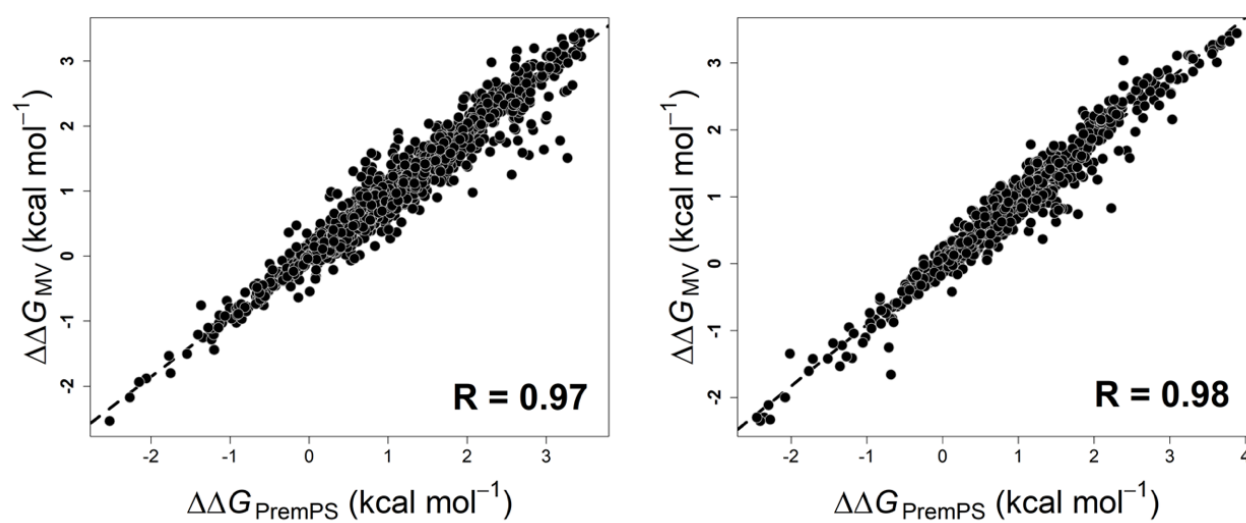

C.

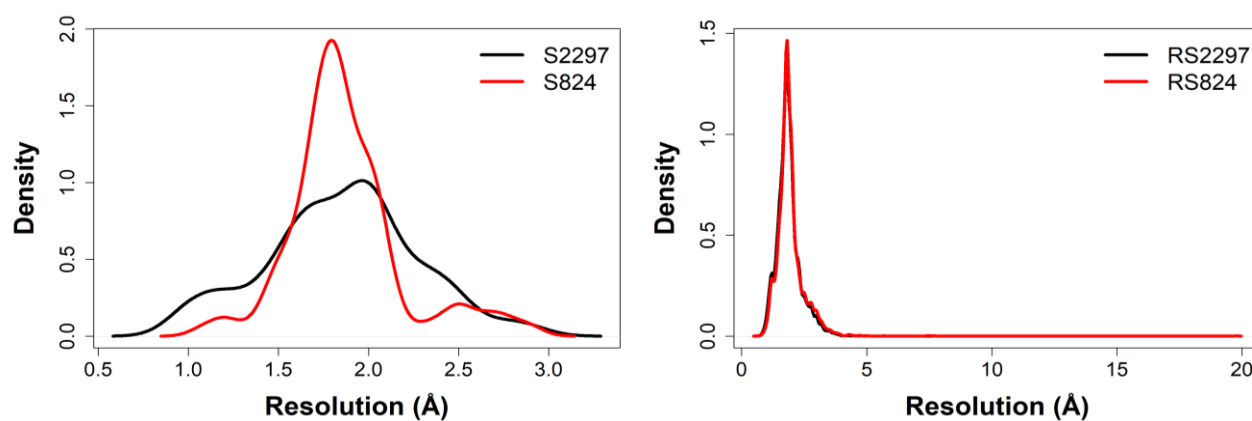

Supplement: S6 Fig — (A) Distribution of mean value (MV) and standard deviation (SD) of ΔΔGPremPS for mutations in datasets of RS2297and RS824. The mean value and standard deviation were calculated using all mapped structures of a protein. (B) Pearson correlation coefficients between ΔΔGPremPS calculated using selected single one structure for a protein in S2297/S824 and the mean value calculated using all other mapped redundant structures in RS2297/RS824. (C) Distribution of resolution of protein structures resolved by X-ray and Cryo-EM. Leave-one-protein-out validation (CV4) results were shown for S2297 and RS2297 datasets. (PDF) [file pcbi.1008543.s006.pdf]

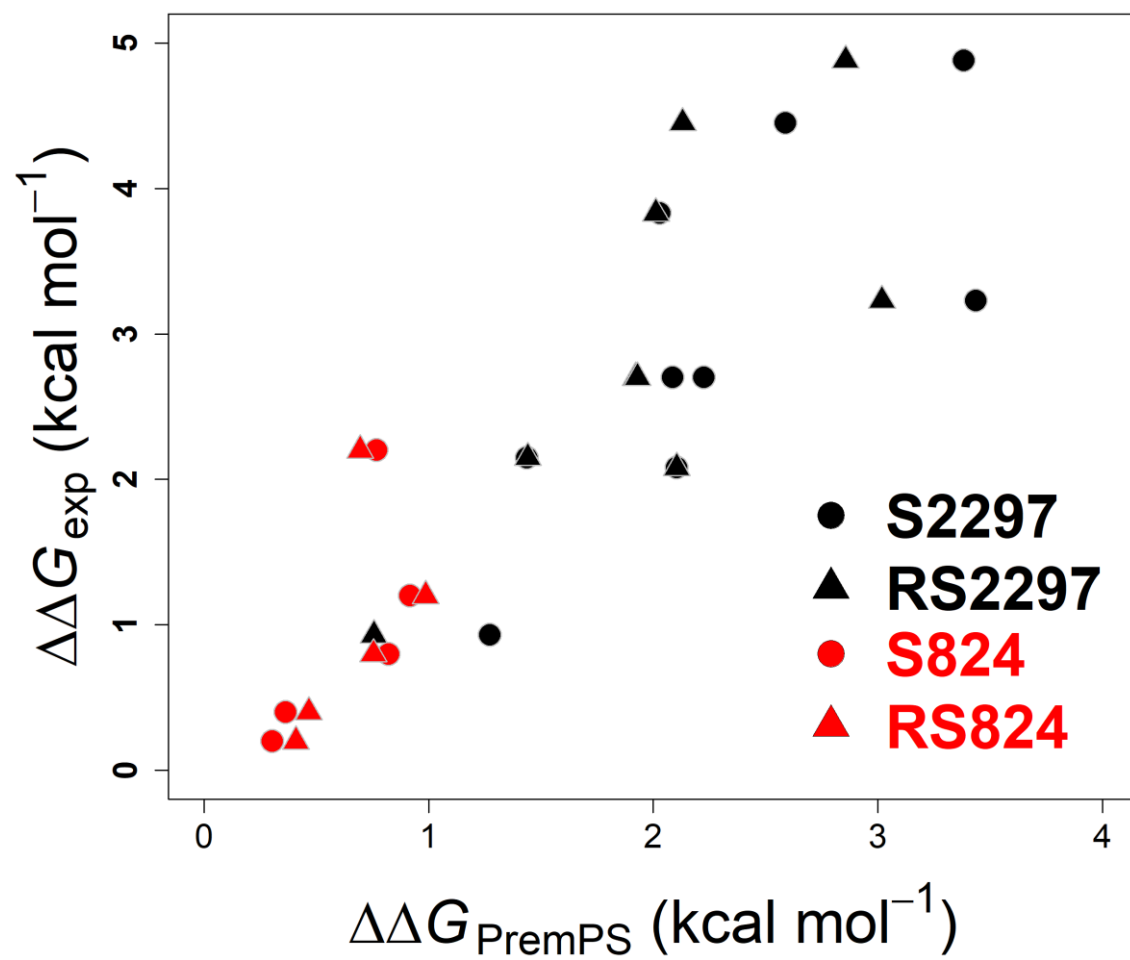

Supplement: S7 Fig — One X-ray and two NMR structures from S2297 and S824 and three structures extracted from high molecular weight Cryo-EM structures (more than 800kDa) from RS2297 and RS824 were used to perform the calculations. Leave-one-protein-out validation (CV4) results are shown for S2297 and RS2297 datasets. The predicted stability changes of four mutations have a relatively large difference of ~ 0.5 kcal mol-1 between using X-ray/NMR structures and two high molecular weight Cryo-EM structures. (PDF) [file pcbi.1008543.s007.pdf]

**A.**

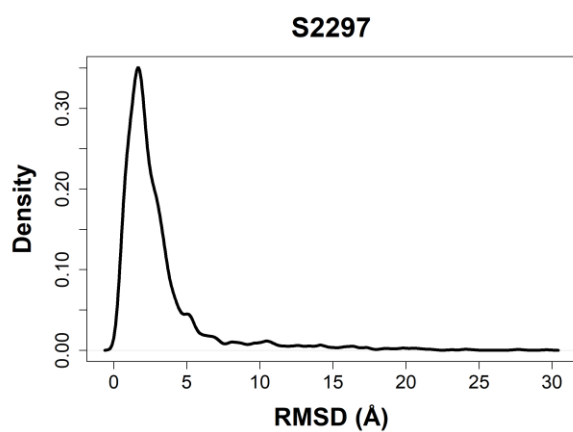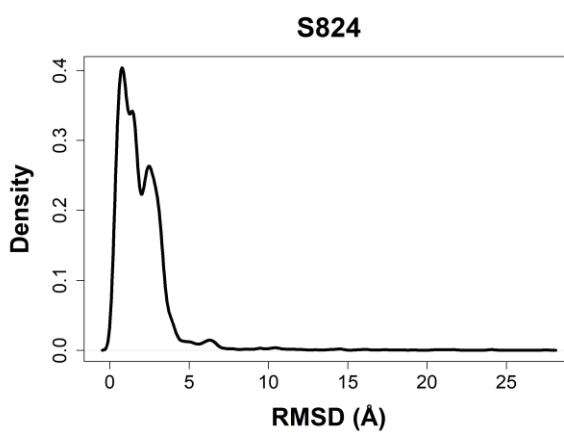

**B.**

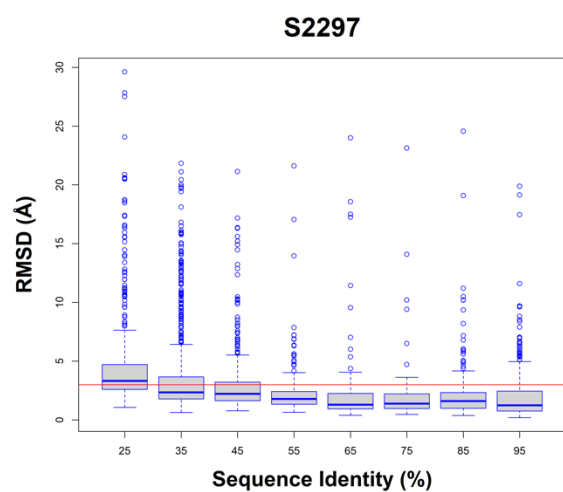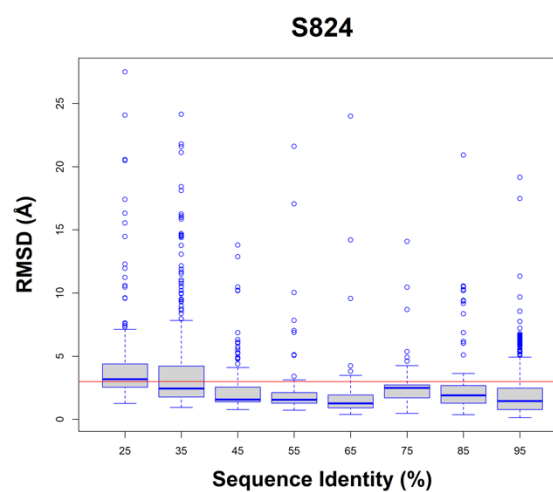

Supplement: S8 Fig — (A) Distribution of root-mean-square deviation (RMSD) between coordinates of all Cα atoms of experimental and modeled structures. (B) Boxplots of RMSD for different ranges of sequence identity of 20–30%, 30–40%…, 90–100%. The red line is 3Å. (PDF) [file pcbi.1008543.s008.pdf]
