# Supplementary material for: PremPS: Predicting the impact of missense mutations on protein stability
Source: PLoS Comput Biol. 2020 Dec 30;16(12):e1008543. doi: 10.1371/journal.pcbi.1008543 (PMC7802934; doi:10.1371/journal.pcbi.1008543)
Supplement: S3 Fig — (A) ROC curves for PremPS trained and tested on S5296 and applying leave-one-protein-out validation (CV4) on S5296. (B) AUC and MCC values for different methods tested on S921. The difference of AUC between PremPS and other methods is significant (p-value << 0.01, DeLong test). Maximum Matthews correlation coefficient is calculated for each method. (C) The definition and the number of mutations for making ROC curves. (PDF) [file pcbi.1008543.s003.pdf]

A. Test on S5296

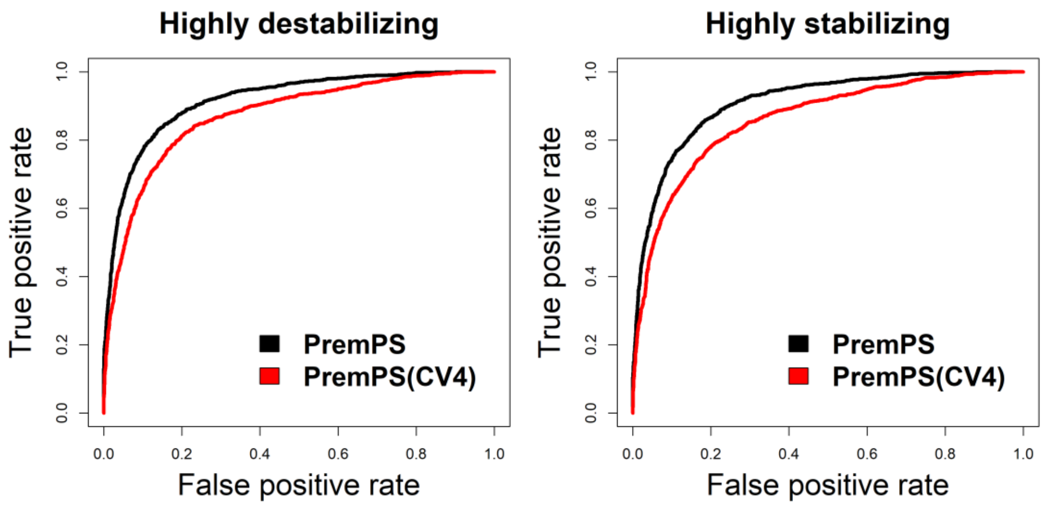

B. Test on S921

Method	Highly destabilizing		Highly stabilizing	
	AUC	MCC	AUC	MCC
PremPS	0.88	0.61	0.87	0.58
INPS3D	0.83	0.54	0.79	0.43
PoPMuSiC	0.81	0.49	0.67	0.21
FoldX	0.80	0.49	0.72	0.35
mCSM	0.75	0.44	0.61	0.14

C.

Category		Definition	# of mutations	
			S5296	S921
**Highly destabilizing**	**Positive**	$\Delta\Delta G_{exp}$ (kcal mol^{-1}) ≥ 1	1364	360
	**Negative**	$\Delta\Delta G_{exp}$ (kcal mol^{-1}) < 1	3932	561
**Highly stabilizing**	**Positive**	$\Delta\Delta G_{exp}$ (kcal mol^{-1}) ≤ -1	1364	109
	**Negative**	$\Delta\Delta G_{exp}$ (kcal mol^{-1}) > -1	3932	812
