# Supplementary material for: PremPS: Predicting the impact of missense mutations on protein stability
Source: PLoS Comput Biol. 2020 Dec 30;16(12):e1008543. doi: 10.1371/journal.pcbi.1008543 (PMC7802934; doi:10.1371/journal.pcbi.1008543)
Supplement: S9 Fig — (A) The entry page of PremPS server. (B) The second step for selecting protein chains. (C) The third step for selecting mutations and three options are provided: “Specify One or More Mutations Manually”, “Upload Mutation List” and “Alanine Scanning for Each Chain”. The mutant structure is produced for each mutation upon the user’s request in the third step. (PDF) [file pcbi.1008543.s009.pdf]

A.

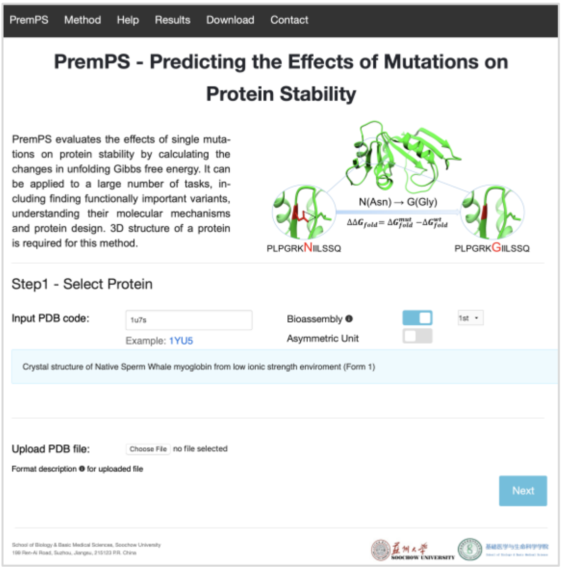
PremPS Method Help Results Download Contact
PremPS - Predicting the Effects of Mutations on Protein Stability
PremPS evaluates the effects of single mutations on protein stability by calculating the changes in unfolding Gibbs free energy. It can be applied to a large number of tasks, including finding functionally important variants, understanding their molecular mechanisms and protein design. 3D structure of a protein is required for this method.
N(Asn) → G(Gly)
PLPGRKNIILSSQ
PLPGRKGIILSSQ
Step1 - Select Protein
Input PDB code:
1u7s
Example: 1YU5
Bioassembly
Asymmetric Unit
1st
Crystal structure of Native Sperm Whale myoglobin from low ionic strength enviroment (Form 1)
Upload PDB file:
Choose File no file selected
Format description for uploaded file
Next

B.

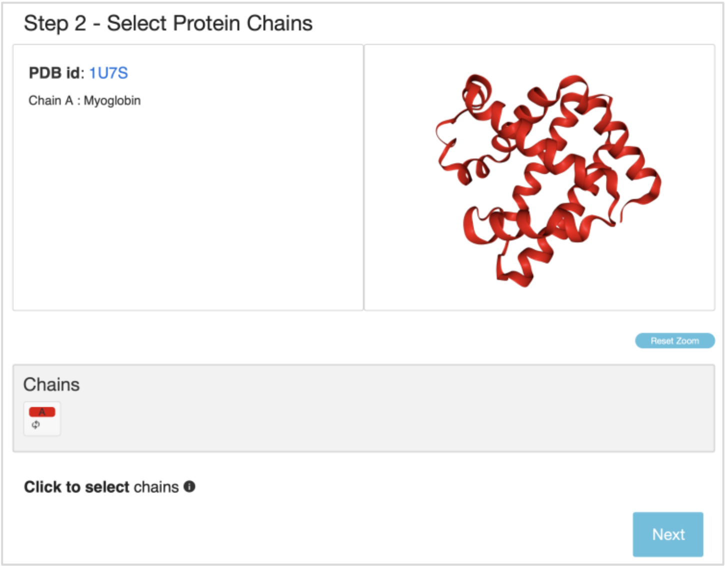
Step 2 - Select Protein Chains
PDB id: 1U7S
Chain A : Myoglobin
Reset Zoom
Chains
A
Click to select chains
Next

C.

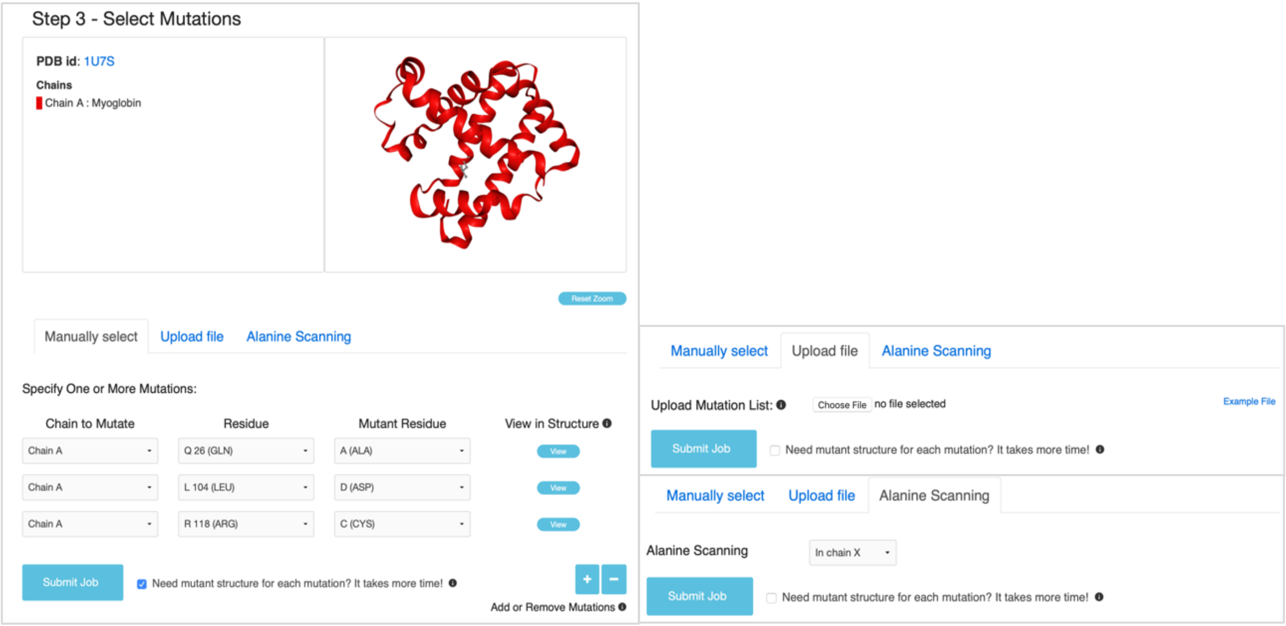
Step 3 - Select Mutations
PDB id: 1U7S
Chains
Chain A : Myoglobin
Reset Zoom
Manually select
Upload file
Alanine Scanning
Specify One or More Mutations:
Chain to Mutate
Residue
Mutant Residue
View in Structure
Chain A
Q 26 (GLN)
A (ALA)
View
Chain A
L 104 (LEU)
D (ASP)
View
Chain A
R 118 (ARG)
C (CYS)
View
Submit Job
Need mutant structure for each mutation? It takes more time!
Add or Remove Mutations
Manually select
Upload file
Alanine Scanning
Upload Mutation List:
Choose File no file selected
Example File
Submit Job
Need mutant structure for each mutation? It takes more time!
Manually select
Upload file
Alanine Scanning
Alanine Scanning
In chain X
Submit Job
Need mutant structure for each mutation? It takes more time!
