# Supplementary material for: PremPS: Predicting the impact of missense mutations on protein stability
Source: PLoS Comput Biol. 2020 Dec 30;16(12):e1008543. doi: 10.1371/journal.pcbi.1008543 (PMC7802934; doi:10.1371/journal.pcbi.1008543)

A.

Job id: 2020060501421741365460632

- Summary

PDB ID	Chains	Number of mutations	Start time (EST)	Processing time	Results
1U7S	A	3	2020-06-04 20:42	5 min	Download

- Results

#	Mutated Chain	Mutation	ΔΔG	Location	Structure
1	A	Q26A	**-0.64**	COR	Explore
2	A	L104D	**1.82**	COR	Explore
3	A	R118C	**0.27**	SUR	Explore

Click

B.

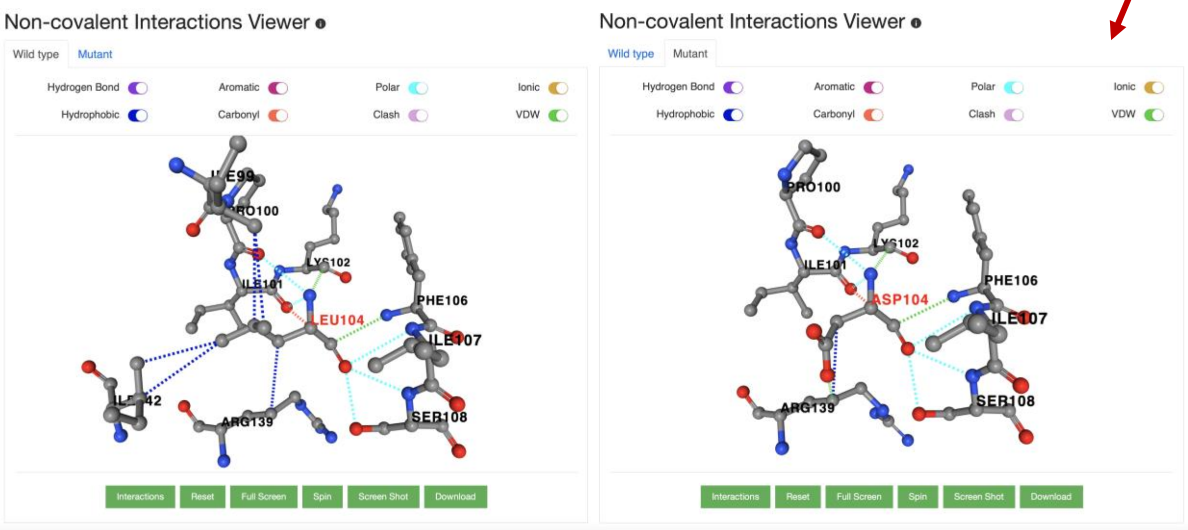

Supplement: S10 Fig — (A) The final results. “Processing time” refers to the running time of a job without counting the waiting time in the queue. The contribution of each feature is provided in the download file. (B) Interactive 3D viewer showing the non-covalent interactions between the mutated site in the protein myoglobin (PDB ID: 1U7S, mutation: L104D) and its adjacent residues in the wild type (left) and mutant (right) respectively, generated by Arpeggio. The mutant structure was produced for each mutation for this job. (PDF) [file pcbi.1008543.s010.pdf]
