# Supplementary material for: PremPS: Predicting the impact of missense mutations on protein stability
Source: PLoS Comput Biol. 2020 Dec 30;16(12):e1008543. doi: 10.1371/journal.pcbi.1008543 (PMC7802934; doi:10.1371/journal.pcbi.1008543)
Supplement: S1 Table — (A) The number of mutations and proteins/structures in each dataset. (B) The number of forward and reverse mutations in the dataset of Ssym, S250 and S2000, respectively. (C) The number of mutations and protein structures (in bracket) in the training dataset of S5296 that overlaps with each test set (the first row) and belongs to the “similar proteins” with more than 25% sequence identity to the proteins in each test set (the second row). (PDF) [file pcbi.1008543.s011.pdf]

A.

Dataset	All mutations			$\Delta\Delta G_{exp} \geq 0$		$\Delta\Delta G_{exp} < 0$	
	# of mutations	# of proteins	# of structures	# of mutations	# of structures	# of mutations	# of structures
**S2648**	2648	129	131	2080	119	568	93
**S5296**	5296	129	131	2682	131	2614	130
**S350**	350	66	67	260	57	90	35
**S605**	605	55	58	467	54	138	34
**S1925**	1925	53	55	1373	48	552	42
**S134**	134	1	6	98	6	36	6
**p53**	42	1	1	31	1	11	1
**S**^{sym}	684	14	357	350	103	334	262
**S250**	250	9	134	125	37	125	101
**S921**	921	54	195	634	82	287	145

One mutation/protein could be mapped to several PDB structures, and the sequences of these structures may be the same or complementary.

B.

Dataset	Forward mutations		Reverse mutations	
	# of mutations	# of structures	# of mutations	# of structures
**S**^{sym}	342	15	342	342
**S250**	125	9	125	125
**S2000**	1000	710	1000	636

C.

**S350**	**S605**	**S1925**	**S134**	**p53**	**S**^{sym}	**S250**	**S2000**	**S921**
350 (67)	277 (40)	903 (45)	41 (1)	5 (1)	402 (14)	218 (9)	228 (13)	0(0)
4334 (75)	3694 (56)	3842 (56)	82 (1)	10 (1)	2444 (18)	1342 (13)	3356 (50)	3710 (47)
