# Supplementary material for: PremPS: Predicting the impact of missense mutations on protein stability
Source: PLoS Comput Biol. 2020 Dec 30;16(12):e1008543. doi: 10.1371/journal.pcbi.1008543 (PMC7802934; doi:10.1371/journal.pcbi.1008543)
Supplement: S2 Table — IncNodePurity is used for describing the importance which is the total decrease in node impurities from splitting on the variable, averaged over all trees. (PDF) [file pcbi.1008543.s012.pdf]

**Feature**	**Importance**
$\boldsymbol{\Delta CS}$	4909
$\boldsymbol{\Delta OMH}$	2502
$\boldsymbol{PSSM}$	2398
$\boldsymbol{SASA_{pro}}$ **and** $\boldsymbol{SASA_{sol}}$	2605
$\boldsymbol{P_{FWY}}$, $\boldsymbol{P_{RKDE}}$ **and** $\boldsymbol{P_{L}}$	2544
$\boldsymbol{N_{Hydro}}$ **and** $\boldsymbol{N_{Charg}}$	1228
