# Supplementary material for: PremPS: Predicting the impact of missense mutations on protein stability
Source: PLoS Comput Biol. 2020 Dec 30;16(12):e1008543. doi: 10.1371/journal.pcbi.1008543 (PMC7802934; doi:10.1371/journal.pcbi.1008543)
Supplement: S3 Table — Leave-one-protein-out validation (CV4) results were shown for S5296. (PDF) [file pcbi.1008543.s013.pdf]

Algorithm	Test set	All mutations			$\Delta\Delta G_{exp} \geq 0$		$\Delta\Delta G_{exp} < 0$	
		**R**	**RMSE**	**Slope**	**R**	**RMSE**	**R**	**RMSE**
**RF**	S5296	0.73	1.23	1.04	0.54	1.20	0.50	1.25
	S921	0.78	1.48	1.52	0.72	1.54	0.60	1.33
**SVM**	S5296	0.70*	1.27	0.97	0.52*	1.26	0.49	1.29
	S921	0.73*	1.53	1.34	0.67*	1.59	0.55*	1.39
**XGBoost**	S5296	0.71*	1.25	0.99	0.53*	1.22	0.50	1.28
	S921	0.77	1.45	1.40	0.71	1.51	0.59	1.32

R: Pearson correlation coefficient between experimental and predicted $\Delta\Delta G$ values. RMSE (kcal mol^{-1}): root-mean-square error. Slope: the slope of the regression line between experimental and predicted $\Delta\Delta G$ values. All presented values of correlation coefficients are statistically significantly different from zero (p-value << 0.01, t-test). *p-value < 0.01 compared to Random Forest (Hittner2003 test).
