# Supplementary material for: PremPS: Predicting the impact of missense mutations on protein stability
Source: PLoS Comput Biol. 2020 Dec 30;16(12):e1008543. doi: 10.1371/journal.pcbi.1008543 (PMC7802934; doi:10.1371/journal.pcbi.1008543)
Supplement: S4 Table — (PDF) [file pcbi.1008543.s014.pdf]

Method	All mutations			$\Delta\Delta G_{exp} \geq 0$		$\Delta\Delta G_{exp} < 0$		Core		Surface	
	R	RMSE	Slope	R	RMSE	R	RMSE	R	RMSE	R	RMSE
PremPS	0.82	1.03	1.08	0.67	1.01	0.63	1.04	0.85	1.15	0.74	0.88
PremPS (CV1)	0.81	1.05	1.07	0.65	1.04	0.62	1.06	0.84	1.17	0.72	0.90
PremPS (CV2)	0.80	1.09	1.08	0.63	1.07	0.60	1.10	0.83	1.21	0.70	0.93
PremPS (CV3)	0.74	1.21	1.10	0.56	1.20	0.54	1.22	0.78	1.34	0.62	1.03
PremPS (CV4)	0.73	1.23	1.04	0.54	1.20	0.50	1.25	0.77	1.37	0.58	1.06
PremPS (CV5)	0.73	1.23	1.03	0.54	1.20	0.50	1.26	0.77	1.37	0.58	1.06

R: Pearson correlation coefficient between experimental and predicted $\Delta\Delta G$ values. RMSE (kcal mol^{-1}): root-mean square error. Slope: the slope of the regression line between experimental and predicted $\Delta\Delta G$ values. All presented values of correlation coefficients are statistically significantly different from zero (p-value << 0.01, t-test). Core/Surface: mutations occur in the protein core/surface.

Method	Forward mutations		Reverse mutations			
	R	RMSE	R	RMSE	R_{RF}	<δ>
PremPS	0.73	1.01	0.71	1.05	-0.92	0.005
PremPS (CV1)	0.72	1.02	0.69	1.06	-0.92	0
PremPS (CV2)	0.69	1.06	0.67	1.10	-0.92	0.001
PremPS (CV3)	0.66	1.17	0.65	1.19	-0.92	0.001
PremPS (CV4)	0.57	1.21	0.54	1.24	-0.90	0.024
PremPS (CV5)	0.57	1.21	0.54	1.25	-0.90	0.021

R_{FR} is the Pearson correlation coefficient between predicted $\Delta\Delta G$ values of the forward and reverse mutations. <δ> is the average bias (= $\sum(\Delta\Delta G_F + \Delta\Delta G_R)$/N; N = 2648, the number of pairs). A non-biased prediction should have R_{FR} and <δ> equal to -1 and 0, respectively.
