# Supplementary material for: PremPS: Predicting the impact of missense mutations on protein stability
Source: PLoS Comput Biol. 2020 Dec 30;16(12):e1008543. doi: 10.1371/journal.pcbi.1008543 (PMC7802934; doi:10.1371/journal.pcbi.1008543)
Supplement: S5 Table — (A), S605 (B), S1925 (C), S134 (D), p53 (E), Ssym (F), S250 (G) and S2000 (H), respectively. The values of R and RMSE for all methods except PremPS were taken from the published papers directly. The performance of PremPS using the same protocol as the other methods when applied to each dataset is shown in bold. In addition, we provided the performance of PremPSM and PremPSP. PremPSM: the model was retrained after removing the overlapped mutations and their corresponding reverse mutations with each test set from the training dataset; PremPSP: the model was retrained after removing all mutations in the “similar proteins” from the training dataset. The number of mutations removed were provided in the S1C Table. (PDF) [file pcbi.1008543.s015.pdf]

A. All methods were retrained after removing S350 from their training sets.

Method	# of predictions	350		309		87	
		R	RMSE	R	RMSE	R	RMSE
**PremPS^M**	**350**	**0.72**	**1.09**	**0.74**	**1.09**	**0.81**	**1.52**
mCSM	350	0.73$^\#$	1.08	0.74$^\#$	1.10	0.82$^\#$	1.48
MAESTRO	350	0.70$^\#$	1.13	0.69$^\#$	1.17	0.76$^\#$	1.67
PoPMuSiC v2.0	350	0.67$^\#$	1.16	0.67$^\#$	1.19	0.71$^\#$	1.67
PoPMuSiC v1.0	350	0.62	1.24	0.63	1.25	0.70$^\#$	1.66
SDM2	350	0.61	1.29	0.61	1.32	0.69$^\#$	1.71
SDM	350	0.52	1.80	0.53	1.81	0.63	2.11
Dmutant	350	0.48	1.81	0.47	1.87	0.57	2.31
AUTOMUTE	315	0.46	1.43	0.45	1.46	0.45	1.99
CUPSAT	346	0.37	1.91	0.35	1.96	0.50	2.14
Eris	334	0.35	4.12	0.34	4.28	0.49	3.91
I-Mutant v2.0	346	0.29	1.65	0.27	1.69	0.27	2.39
PremPS^P	*350*	*0.58*	*1.28*	*0.59*	*1.30*	*0.60*	*1.94*

The values of R and RMSE for other methods except PremPS were taken from [21, 22, 24, 25] directly. 350 mutations were tested using each method, while some methods failed to compute the $\Delta\Delta G$ for some mutations, so the predicted $\Delta\Delta G$ values were set to zero when counted these mutations. 309 mutations for which the $\Delta\Delta G$ values are available for all methods, and among them 87 mutations whose experimental $|\Delta\Delta G|$ are ≥ 2 kcal mol^{-1}.

The differences in R between PremPS (in bold) and other methods are significant except $^\#$p-value > 0.05 (Fisher1925 test).

B. PremPS (in bold) and other methods except Meta-predictor were applied to the dataset of S605 directly. S605 is the training set of Meta-predictor, and the R and RMSE reported by Meta-predictor are the mean values across 1000 tests. Namely, Meta-predictor randomly chose 50% mutations from S605 as training and used the remaining mutations for testing; the procedure was repeated 1000 times.

Method	R	RMSE
**PremPS**	**0.80**	**1.34**
Meta-predictor	0.73	1.29
PoPMuSiC v2.0	0.68	1.32
DFire	0.64	1.84
CUPSAT	0.55	1.77
FoldX	0.54	1.78
Rosetta	0.54	2.34
MultiMutate	0.54	2.34
EGAD	0.52	1.61
I-Mutant v3.0	0.51	1.52
MUPRO	0.49	1.52
SDM	0.46	1.96
Hunter	0.32	1.89
PremPS^M	*0.70*	*1.51*
PremPS^P	*0.62*	*1.71*

The values of R and RMSE for other methods were taken from [26].
The differences in R between PremPS and all other methods are significant (p-value < 0.01, Fisher1925 test).

C. S1925 is the training dataset of AUTOMUTE. All methods were retrained on this dataset and the R and RMSE are the results of 20-fold cross-validation on S1925.

Method	R	RMSE
**PremPS**	**0.87**	**0.90**
mCSM	0.82	1.00
AUTOMUTE (REPTree)	0.79	1.10
AUTOMUTE (SVMreg)	0.76	1.20
I-Mutant v2.0	0.71	1.30

The values of R and RMSE for other methods were taken from [22].
The differences in R between PremPS and other methods are significant (p-value < 0.01, Fisher1925 test).

D. Pearson correlation coefficients between experimental and predicted $\Delta\Delta G$ values for different methods applied on 134 mutations from six high-resolution structures of myoglobin (PDB IDs are shown in the first row). All methods were applied to this dataset directly. AVR: correlation coefficient between experimental and average values of the six outputs.

Method	1A6G	1A6M	1BZ6	1BZP	1U7S	2EKT	AVR
**PremPS**	**0.71**	**0.72**	**0.73**	**0.73**	**0.75**	**0.73**	**0.73**
I-Mutant v2.0	0.65#	0.65#	0.64#	0.65#	0.64#	0.65#	0.65#
SDM	0.58#	0.58	0.60#	0.57	0.60	0.59	0.59
PoPMuSiC v2.1	0.54	0.55	0.56	0.57	0.55	0.55	0.56
I-Mutant v3.0	0.54	0.54	0.55	0.54	0.53	0.54	0.54
mCSM	0.35	0.39	0.40	0.44	0.47	0.44	0.44
CUPSAT	0.36	0.31	0.25	0.30	0.45	0.48	0.40
$PremPS^M$	*0.65#*	*0.65#*	*0.65#*	*0.65#*	*0.65#*	*0.64#*	*0.65#*
$PremPS^P$	*0.65#*	*0.65#*	*0.65#*	*0.65#*	*0.65#*	*0.64#*	*0.65#*

The values of R and RMSE for other methods were taken from [49].

The differences in R between PremPS and other methods are significant except #p-value > 0.05 (Fisher1925 test).

E. All methods were tested on the dataset of p53 directly.

Method	R	RMSE
**PremPS**	**0.73**	**1.41**
DUET	0.68	1.39
mCSM	0.68	1.40
PoPMuSiC v2.0	0.56	1.52
SDM	0.52	1.61
iStable	0.49	1.59
$PremPS^M$	*0.72*	*1.47*
$PremPS^P$	*0.72*	*1.47*

The values of R and RMSE for other methods were taken from [22, 23].

The difference in R between PremPS and other methods is not significant (p-value > 0.05, Fisher1925 test).

F. All methods were applied to the dataset of S^{sym} directly.

Method	Forward mutations		Reverse mutations			
	R	RMSE	R	RMSE	R_{FR}	<δ>
**PremPS**	**0.81**	**0.96**	**0.74**	**1.12**	**-0.93**	**0.03**
DDGun3D	0.56	1.42	0.53	1.46	-0.99	-0.02
INPS	0.51	1.42	0.50	1.44	-0.99	-0.04
DDGun	0.48	1.47	0.48	1.50	-0.99	-0.01
$PoPMuSiC^{sym}$	0.48	1.58	0.48	1.62	-0.77	0.03
Blind-INPS	0.48	1.44	0.47	1.45	-0.99	-0.06
INPS3D	0.59	1.29	0.44	1.64	-0.86	-0.55
Rosetta	0.69	2.31	0.43	2.61	-0.41	-0.69
FoldX	0.63	1.56	0.39	2.13	-0.38	-0.47
MAESTRO	0.52	1.36	0.32	2.09	-0.34	-0.58
SDM	0.51	1.74	0.32	2.28	-0.75	-0.32
PoPMuSiC v2.1	0.63	1.21	0.25	2.18	-0.29	-0.71
mCSM	0.61	1.23	0.14	2.43	-0.26	-0.91
DUET	0.63	1.20	0.13	2.38	-0.21	-0.84
MUPRO	$0.79^{\#}$	0.94	0.07	2.51	-0.02	-0.97
CUPSAT	0.39	1.71	0.05	2.88	-0.54	-0.72
NeEMO	0.72	1.08	0.02	2.35	0.09	-0.60
AUTOMUTE	0.73	1.07	-0.01	2.61	-0.06	-0.99
I-Mutant v3.0	0.62	1.23	-0.04	2.32	0.02	-0.68
iStable	0.72	1.10	-0.08	2.28	-0.05	-0.60
STRUM	0.75	1.05	-0.15	2.51	0.34	-0.87
$PremPS^{M}$	*0.64*	*1.21*	*0.56*	*1.30*	*$-0.91^{\#}$*	*0.03*
$PremPS^{P}$	*0.56*	*1.32*	*0.50*	*1.37*	*-0.89*	*0.04*

The values of R and RMSE for other methods were taken from [48, 55, 57].

R_{FR} is the Pearson correlation coefficient between predicted $\Delta\Delta G$ values of the forward and reverse mutations. <δ> = $\sum(\Delta\Delta G_F + \Delta\Delta G_R)$/N. A non-biased prediction should have R_{FR} = -1 and <δ> = 0.

The differences in R between PremPS and other methods are significant except #p-value > 0.05 (Fisher1925 test). The methods are ranked according to the R of reverse mutations.

G. All methods were applied to the dataset of S250 directly.

Mehod	R	R_F	R_R	R_{FR}	<δ>	Inconsistency
**PremPS**	**0.89**	**0.87**	**0.82**	**-0.94**	**0.04**	**12**
INPS	0.67	0.51	0.51	-0.99	-0.01	3.2
I-Mutant v2.0	0.60	0.94	0.05	-0.09	-2.10	77.6
mCSM	0.47	0.65	-0.04	-0.15	-1.66	80.8
MUPRO	0.57	0.97	-0.02	0.05	-1.85	73.6
DUET	0.48	0.65	-0.02	-0.11	-1.54	73.6
STRUM	0.60	0.84[#]	-0.06	0.06	-1.38	75.2
$PremPS^M$	*0.78*	*0.68*	*0.61*	*-0.92*[#]	*-0.05*	*7.2*
$PremPS^P$	*0.74*	*0.60*	*0.56*	*-0.88*	*-0.04*	*4*

The values of R and RMSE for other methods were taken from [58,59].

R, R_F and R_R is the Pearson correlation coefficient between experimental and predicted $\Delta\Delta G$ values for all, forward and reverse mutations, respectively. R_{FR} is the Pearson correlation coefficient between predicted $\Delta\Delta G$ values of the forward and reverse mutations. <δ> = $\sum(\ \Delta\Delta G_F + \Delta\Delta G_R\ )$/N. Inconsistency = the percentage of forward mutations and their reverse pairs predicted with the same sign.

The differences in R between PremPS and other methods are significant except [#]p-value > 0.05 (Fisher1925 test). The methods are ranked according to the correlation coefficient of reverse mutations.

H. All methods were applied to the dataset of S2000 directly.

Method	R_{RF}	<δ>/2 ± SE
**PremPS**	**-0.92**	**0.05 ± 0.01**
INPS	-0.95	0.04 ± 0.15
INPS3D	-0.82	0.29 ± 0.27
Eris	-0.39	1.25 ± 0.11
FoldX	-0.15	0.74 ± 0.05
I-Mutant v2.0	-0.13	0.80 ± 0.01
Rosetta	-0.06	2.08 ± 0.12
$PremPS^M$	*-0.92*[#]	*0.04 ± 0.01*
$PremPS^P$	*-0.89*	*0.07 ± 0.01*

The values of R and RMSE for other methods were taken from [55, 56].

SE: standard error.

The differences in R between PremPS and other methods are significant except [#]p-value > 0.05 (Fisher1925 test).
