# Supplementary material for: PremPS: Predicting the impact of missense mutations on protein stability
Source: PLoS Comput Biol. 2020 Dec 30;16(12):e1008543. doi: 10.1371/journal.pcbi.1008543 (PMC7802934; doi:10.1371/journal.pcbi.1008543)
Supplement: S6 Table — PremPS: the model was trained on S5296; PremPSF: the model was trained on the forward mutation dataset of S2648; PremPSP: the model was retrained after removing all mutations in the “similar proteins” with more than 25% sequence identity to the proteins in S921 from the training dataset. (PDF) [file pcbi.1008543.s016.pdf]

Method	All mutations			$\Delta\Delta G_{exp} \geq 0$		$\Delta\Delta G_{exp} < 0$		Core		Surface	
	R	RMSE	Slope	R	RMSE	R	RMSE	R	RMSE	R	RMSE
PremPS	0.78	1.48	1.52	0.72	1.54	0.60	1.33	0.82	1.81	0.55	1.11
PremPS[F]	0.73	1.60	1.76	0.73[a]	1.51	0.29	1.79	0.80[b]	2.00	0.50	1.15
PremPS[P]	0.70	1.69	1.68	0.59	1.83	0.60[c]	1.31	0.75	2.13	0.48	1.17
INP3D	0.68	1.62	1.40	0.64	1.61	0.38	1.64	0.74	2.00	0.44	1.19
INPS	0.66	1.66	1.23	0.57	1.76	0.46	1.42	0.71	2.07	0.46	1.19
PoPMuSiC	0.64	1.68	1.30	0.68[d]	1.48	-	2.06	0.70	2.09	0.39	1.22
FoldX	0.57	2.06	0.54	0.56	1.99	0.22	2.21	0.58	2.58	0.41	1.45
mCSM	0.52	1.85	1.26	0.57	1.63	-	2.25	0.60	2.28	0.17	1.36

Only correlation coefficients with statistically significantly different from zero (p-value < 0.01, t-test) are shown. The differences in R between PremPS and other methods are significant (p-values < 0.01, Hittner2003 test) except [a]p-value = 0.27, [b]p-value = 0.02, [c]p-value = 0.84, and [d]p-value = 0.04.

The differences in R between INPS3D and INPS are not significant (p-value > 0.1) except for destabilizing mutations with p-value < 0.01, so the INPS3D presents better performance than INPS on S921 dataset.
