# Supplementary material for: PremPS: Predicting the impact of missense mutations on protein stability
Source: PLoS Comput Biol. 2020 Dec 30;16(12):e1008543. doi: 10.1371/journal.pcbi.1008543 (PMC7802934; doi:10.1371/journal.pcbi.1008543)
Supplement: S7 Table — (A) The number of mutations from monomeric and homomeric protein structures in S2648 dataset, respectively. The number of protein structures are shown in parentheses. Interface: mutations at the protein-protein interface of homomers. (B) The number of monomeric protein structures/mutations that can be mapped to multiple experimental structures in S2648 and S921, respectively. The number of mapped redundant structures has excluded the selected structures used in the datasets of S2648 and S921. (C) The number of protein structures resolved by experimental method of X-ray, NMR or Cryo-EM and two or three methods. (D) The number of protein structures resolved at different resolutions. (E) The number of protein structures resolved in a monomeric state or extracted from homomers and heteromers. (F) The number of proteins for which at least one templates were found and the number of modeled structures in each range of sequence identity. (G) The number of proteins and modeled structures in each range of root-mean-square deviation. (PDF) [file pcbi.1008543.s017.pdf]

A.

Monomer	Homomer		
	All mutations	Interface	Non-interface
2372 (104)	276 (27)	135 (19)	141 (21)

B.

Dataset	# of proteins (# of mutations)	# of mapped redundant structures (# of mapped mutations)
S2648	99 (2297)	6012 (331648)
S921	103 (824)	4429 (172253)

C.

Method	# of proteins (# of muts)	# of mapped structs (# of mapped muts)	# of proteins (# of muts)	# of mapped structs (# of mapped muts)
	**S2297**		**S824**	
X-ray	82 (2088)		96 (760)	
NMR	17 (209)		7 (64)	
	**RS2297**		**RS824**	
X-ray	90 (2227)	5667 (322671)	102 (795)	4127 (169138)
NMR	68 (1781)	319 (8223)	49 (741)	269 (2998)
Cryo-EM	8 (157)	26 (754)	9 (50)	33 (117)
**X-ray, NMR and Cryo-EM**				
X-ray		1249 (51061)		1258 (3039)
NMR	7 (94)	92 (1008)	8 (43)	127 (431)
Cryo-EM		25 (691)		32 (110)
**X-ray and NMR**				
X-ray	59 (1711)	5011 (306441)	48 (715)	4047 (166405)
NMR		285 (8042)		264 (2878)
**X-ray and Cryo-EM**				
X-ray	8 (157)	1252 (51250)	9 (46)	1261 (3048)
Cryo-EM		26 (754)		33 (113)
**NMR and Cryo-EM**				
NMR	7 (94)	92 (1008)	8 (44)	127 (432)
Cryo-EM		25 (691)		32 (111)

D.

Resolution	# of proteins (# of muts)	# of mapped structs (# of mapped muts)	# of proteins (# of muts)	# of mapped structs (# of mapped muts)
	**S2297**		**S824**	
≤ 3Å	82 (2088)		96 (760)	
≤ 2Å	60 (1607)		78 (596)	
2Å ~ 3Å	22 (481)		18 (164)	
	**RS2297**		**RS824**	
≤ 3Å	89 (2225)	5533 (318413)	101 (794)	3993 (162479)
> 3Å	28 (518)	157 (4939)	27 (435)	164 (6768)
≤ 2Å	80 (2176)	4178 (262487)	94 (782)	2967 (124944)
2Å ~ 3Å	77 (2050)	1355 (55926)	67 (683)	1026 (37535)
3Å ~ 4Å	26 (453)	146 (4737)	25 (402)	147 (6487)
> 4Å	9 (194)	11 (202)	11 (262)	17 (281)
**≤ 3Å and > 3Å**				
≤ 3Å	27 (516)	2972 (132705)	26 (430)	2886 (140969)
> 3Å		156 (4937)		164 (6763)
**≤ 2Å, 2Å ~ 3Å, 3Å ~ 4Å and > 4Å**				
≤ 2Å	7 (129)	822 (47424)	9 (229)	821 (84838)
2Å ~ 3Å		478 (17901)		486 (30974)
3Å ~ 4Å		90 (3541)		95 (6134)
> 4Å		9 (137)		15 (248)

E.

State	# of proteins (# of muts)	# of mapped structs (# of mapped muts)	# of proteins (# of muts)	# of mapped structs (# of mapped muts)
	**S2297**		**S824**	
Monomer	68 (1760)		82 (600)	
Homomer	25 (501)		20 (200)	
Heteromer	6 (36)		1 (24)	
	**RS2297**		**RS824**	
Monomer	90 (2160)	3841 (269705)	95 (812)	2723 (136629)
Homomer	71 (2101)	1382 (42214)	65 (716)	1099 (24486)
Heteromer	49 (1564)	789 (19729)	33 (489)	607 (11138)
**Monomers, homomers and heteromers**				
Monomer	35 (1462)	2326 (248857)	30 (450)	2212 (114594)
Homomer		1077 (36894)		1008 (23036)
Heteromer		653 (18476)		579 (10803)

F.

Sequence identity	# of proteins (# of muts)	# of modeled structs (# of mapped muts)	# of proteins (# of muts)	# of modeled structs (# of mapped muts)
	**S2297**		**S824**	
20-30%	37 (607)	415 (7617)	19 (172)	211 (6071)
30-40%	68 (1246)	824 (13810)	35 (314)	369 (3044)
40-50%	57 (1128)	417 (7184)	75 (503)	264 (2235)
50-60%	44 (771)	177 (3307)	26 (212)	102 (582)
60-70%	46 (939)	120 (2910)	31 (371)	76 (656)
70-80%	41 (803)	102 (2235)	69 (466)	119 (893)
80-90%	44 (923)	173 (3306)	28 (369)	153 (2185)
90-100%	87 (2131)	561 (17802)	99 (780)	4797 (25598)

G.

RMSD	# of proteins (# of muts)	# of modeled structs (# of mapped muts)	# of proteins (# of muts)	# of modeled structs (# of mapped muts)
	**S2297**		**S824**	
≤ 3Å	90 (2144)	1926 (42012)	101 (791)	5106 (32764)
3Å ~ 5Å	71 (1308)	494 (9571)	82 (555)	755 (7098)
5Å ~ 10Å	50 (997)	224 (2698)	74 (439)	167 (1035)
> 10Å	45 (716)	145 (2890)	17 (174)	63 (367)
