# Supplementary material for: PremPS: Predicting the impact of missense mutations on protein stability
Source: PLoS Comput Biol. 2020 Dec 30;16(12):e1008543. doi: 10.1371/journal.pcbi.1008543 (PMC7802934; doi:10.1371/journal.pcbi.1008543)
Supplement: S8 Table — (PDF) [file pcbi.1008543.s018.pdf]

Multimeric state	Method	All mutations		Interface		Non-interface	
		**R**	**RMSE**	**R**	**RMSE**	**R**	**RMSE**
Homomer	PremPS	0.95	0.46	0.95	0.41	0.96	0.50
	PremPS (CV4)	0.56	1.08	0.62	0.96	0.51	1.18
Monomer extracted from homomer	PremPS	0.68*	0.98	0.64*	1.06	0.77*	0.90
	PremPS (CV4)	0.47*	1.17	0.49*	1.15	0.51	1.18

*p-value < 0.01 compared to homomer (Hittner2003 test).
