# Supplementary material for: PremPS: Predicting the impact of missense mutations on protein stability
Source: PLoS Comput Biol. 2020 Dec 30;16(12):e1008543. doi: 10.1371/journal.pcbi.1008543 (PMC7802934; doi:10.1371/journal.pcbi.1008543)
Supplement: S9 Table — S2297 and S824, subsets of S2648 and S921 respectively, consist of selected single one structure for a protein, while the datasets of RS2297 and RS824 include all the other mapped redundant structures. (PDF) [file pcbi.1008543.s019.pdf]

Dataset	Method	R	RMSE
S2297	PremPS	0.96	0.47
	PremPS (CV4)	0.57	1.23
RS2297	PremPS	0.84*	0.88
	PremPS (CV4)	0.59	1.23
S824	PremPS	0.74	1.48
RS824	PremPS	0.71	1.61

*p-value < 0.01 compared to S2297 (Fisher1925 test).
