# Supplementary material for: PremPS: Predicting the impact of missense mutations on protein stability
Source: PLoS Comput Biol. 2020 Dec 30;16(12):e1008543. doi: 10.1371/journal.pcbi.1008543 (PMC7802934; doi:10.1371/journal.pcbi.1008543)
Supplement: S10 Table — The number of proteins resolved by both NMR and Cryo-EM are almost the same as that resolved by X-ray, NMR and Cryo-EM (see S7C Table), so the performance for two methods of NMR and Cryo-EM is not shown. (PDF) [file pcbi.1008543.s020.pdf]

| | Method | X-ray | | NMR | | Cryo-EM | |
|---|---|---|---|---|---|---|---|
| | | R | RMSE | R | RMSE | R | RMSE |
| S2297 | PremPS | 0.96 | 0.48 | 0.97 | 0.40 | | |
| | PremPS (CV4) | 0.55 | 1.25 | 0.73* | 0.96 | | |
| RS2297 | PremPS | 0.84 | 0.88 | 0.81* | 0.91 | 0.91* | 0.65 |
| | PremPS (CV4) | 0.59 | 1.23 | 0.58 | 1.22 | 0.72* | 1.00 |
| S824 | PremPS | 0.75 | 1.50 | 0.65 | 1.20 | | |
| RS824 | PremPS | 0.71 | 1.61 | 0.73 | 1.72 | 0.64 | 2.37 |
| **X-ray, NMR and Cryo-EM** | | | | | | | |
| RS2297 | PremPS | 0.91 | 0.66 | 0.80* | 0.78 | 0.91 | 0.67 |
| | PremPS (CV4) | 0.72 | 1.01 | 0.51* | 1.08 | 0.72 | 1.02 |
| RS824 | PremPS | 0.42 | 3.50 | 0.90* | 1.86 | 0.64* | 2.43 |
| **X-ray and NMR** | | | | | | | |
| RS2297 | PremPS | 0.84 | 0.89 | 0.81* | 0.91 | | |
| | PremPS (CV4) | 0.60 | 1.24 | 0.57* | 1.23 | | |
| RS824 | PremPS | 0.72 | 1.61 | 0.74 | 1.75 | | |
| **X-ray and Cryo-EM** | | | | | | | |
| RS2297 | PremPS | 0.91 | 0.66 | | | 0.91 | 0.65 |
| | PremPS (CV4) | 0.72 | 1.01 | | | 0.72 | 1.00 |
| RS824 | PremPS | 0.42 | 3.49 | | | 0.64* | 2.40 |

*p-value < 0.01 compared to X-ray (Fisher1925 test).
