# Supplementary material for: PremPS: Predicting the impact of missense mutations on protein stability
Source: PLoS Comput Biol. 2020 Dec 30;16(12):e1008543. doi: 10.1371/journal.pcbi.1008543 (PMC7802934; doi:10.1371/journal.pcbi.1008543)
Supplement: S11 Table — (PDF) [file pcbi.1008543.s021.pdf]

| | **Method** | **≤ 3Å** | | **> 3Å** | | **≤ 2Å** | | **2Å ~ 3Å** | | **3Å ~ 4Å** | | **> 4Å** | |
|---|---|---|---|---|---|---|---|---|---|---|---|---|---|
| | | **R** | **RMSE** | **R** | **RMSE** | **R** | **RMSE** | **R** | **RMSE** | **R** | **RMSE** | **R** | **RMSE** |
| S2297 | PremPS | 0.96 | 0.48 | | | 0.97 | 0.47 | 0.95* | 0.49 | | | | |
| | PremPS (CV4) | 0.55 | 1.25 | | | 0.59 | 1.24 | 0.42* | 1.29 | | | | |
| RS2297 | PremPS | 0.84 | 0.87 | 0.58* | 1.36 | 0.85 | 0.86 | 0.81* | 0.95 | 0.58* | 1.37 | 0.60 | 1.26 |
| | PremPS (CV4) | 0.59 | 1.23 | 0.41* | 1.50 | 0.60 | 1.22 | 0.57* | 1.28 | 0.40* | 1.51 | 0.45 | 1.39 |
| S824 | PremPS | 0.75 | 1.50 | | | 0.76 | 1.36 | 0.74 | 1.93 | | | | |
| RS824 | PremPS | 0.72 | 1.61 | 0.56* | 1.57 | 0.73 | 1.62 | 0.67* | 1.59 | 0.56* | 1.57 | 0.56 | 1.62 |
| | | **Overlapped proteins** | | | | **Overlapped proteins** | | | | | | | |
| RS2297 | PremPS | 0.84 | 0.88 | 0.58* | 1.36 | 0.84 | 1.02 | 0.73* | 1.19 | 0.46* | 1.48 | 0.52 | 1.51 |
| | PremPS (CV4) | 0.60 | 1.22 | 0.41* | 1.50 | 0.54 | 1.37 | 0.48* | 1.44 | 0.34* | 1.55 | 0.38 | 1.61 |
| RS824 | PremPS | 0.65 | 1.36 | 0.56* | 1.57 | 0.69 | 1.32 | 0.65* | 1.38 | 0.56* | 1.55 | 0.55 | 1.69 |

*p-value < 0.01 compared to the previous resolution (Fisher1925 test).
