# Supplementary material for: PremPS: Predicting the impact of missense mutations on protein stability
Source: PLoS Comput Biol. 2020 Dec 30;16(12):e1008543. doi: 10.1371/journal.pcbi.1008543 (PMC7802934; doi:10.1371/journal.pcbi.1008543)
Supplement: S12 Table — (PDF) [file pcbi.1008543.s022.pdf]

	Method	Monomer		Homomer		Heteromer	
		**R**	**RMSE**	**R**	**RMSE**	**R**	**RMSE**
S2297	PremPS	0.96	0.46	0.96	0.51	0.93	0.49
	PremPS (CV4)	0.60	1.16	0.47*	1.44	0.49	1.17
RS2297	PremPS	0.84	0.87	0.83*	0.92	0.84	0.94
	PremPS (CV4)	0.59	1.22	0.58*	1.29	0.62*	1.29
S824	PremPS	0.75	1.47	0.71	1.50	0.73	1.61
RS824	PremPS	0.72	1.57	0.69*	1.81	0.67*	1.69
**Monomers, homomers and heteromers**							
RS2297	PremPS	0.84	0.88	0.83*	0.94	0.84	0.94
	PremPS (CV4)	0.61	1.22	0.58*	1.29	0.61	1.29
RS824	PremPS	0.72	1.66	0.69*	1.84	0.67*	1.69

*p-value < 0.01 compared to monomer (Fisher1925 test).
