# Supplementary material for: PremPS: Predicting the impact of missense mutations on protein stability
Source: PLoS Comput Biol. 2020 Dec 30;16(12):e1008543. doi: 10.1371/journal.pcbi.1008543 (PMC7802934; doi:10.1371/journal.pcbi.1008543)
Supplement: S13 Table — (PDF) [file pcbi.1008543.s023.pdf]

Dataset	Structure	Method	20-30%	30-40%	40-50%	50-60%	60-70%	70-80%	80-90%	90-100%
**Pearson correlation coefficient (R)**										
S2297	Exp. Structs.	PremPS	0.96	0.96	0.96	0.96	0.96	0.96	0.96	0.96
		PremPS (CV4)	0.62	0.58	0.57	0.55	0.56	0.58	0.53	0.57
	All models	PremPS	0.68*	0.78*	0.81*	0.80*	0.85*	0.86*	0.84*	0.85*
		PremPS (CV4)	0.49*	0.53	0.54	0.50	0.56	0.60	0.57	0.53
	Selected models	PremPS	0.76*	0.81*	0.83*	0.83*	0.84*	0.85*	0.85*	0.88*
		PremPS (CV4)	0.56	0.55	0.56	0.55	0.54	0.57	0.53	0.58
S824	Exp. Structs.	PremPS	0.72	0.68	0.69	0.57	0.72	0.74	0.70	0.74
	All models	PremPS	0.65	0.67	0.71	0.57	0.67	0.69	0.68	0.76
	Selected models	PremPS	0.55*	0.58	0.66	0.57	0.74	0.72	0.66	0.72
**Root-mean-square error (RMSE)**										
S2297	Exp. Structs.	PremPS	0.47	0.49	0.50	0.47	0.50	0.49	0.49	0.47
		PremPS (CV4)	1.20	1.27	1.28	1.24	1.33	1.27	1.30	1.24
	All models	PremPS	1.02	1.00	1.00	0.94	0.83	0.77	0.79	0.88
		PremPS (CV4)	1.24	1.31	1.36	1.32	1.23	1.15	1.16	1.31
	Selected models	PremPS	1.02	0.96	0.92	0.86	0.91	0.88	0.86	0.78
		PremPS (CV4)	1.27	1.30	1.30	1.24	1.34	1.28	1.30	1.23
S824	Exp. Structs.	PremPS	1.59	1.41	1.38	1.46	1.35	1.36	1.59	1.51
	All models	PremPS	1.01	1.59	1.51	1.54	1.46	1.59	1.52	1.34
	Selected models	PremPS	1.76	1.52	1.42	1.46	1.35	1.38	1.66	1.53

*p-value < 0.01 compared to experimental structures (Fisher1925 test).
