# Supplementary material for: PremPS: Predicting the impact of missense mutations on protein stability
Source: PLoS Comput Biol. 2020 Dec 30;16(12):e1008543. doi: 10.1371/journal.pcbi.1008543 (PMC7802934; doi:10.1371/journal.pcbi.1008543)
Supplement: S14 Table — (PDF) [file pcbi.1008543.s024.pdf]

| Dataset | Method | ≤ 3Å | | 3Å~5Å | | 5Å~10Å | | > 10Å | |
|---|---|---|---|---|---|---|---|---|---|
| | | R | RMSE | R | RMSE | R | RMSE | R | RMSE |
| S2272 | PremPS | 0.83 | 0.88 | 0.77* | 1.04 | 0.78 | 1.07 | 0.57* | 1.21 |
| | PremPS (CV4) | 0.54 | 1.25 | 0.50* | 1.38 | 0.52 | 1.41 | 0.36* | 1.38 |
| S824 | PremPS | 0.74 | 1.34 | 0.70* | 1.32 | 0.59* | 1.87 | 0.44* | 1.51 |

*p-value < 0.01 compared to the previous RMSD (Fisher1925 test).
